# Phylum-wide propionate degradation and its potential connection to poly-gamma-glutamate biosynthesis in *Candidatus* Cloacimonadota phylum

**DOI:** 10.1093/ismejo/wrag055

**Published:** 2026-03-18

**Authors:** Magdalena Calusinska, Malte Herold, Dominika Klimek, Marie Bertucci, Sébastien Lemaigre, Sébastien Cambier, Simone Zorzan, Céline Leclercq, Jan Dolfing, Maria Westerholm, Bettina Müller, Leila Nasirzadeh, Anna Schnürer, Paul Wilmes, Philippe Delfosse, Xavier Goux

**Affiliations:** Environmental and Industrial Biotechnologies, Luxembourg Institute of Science and Technology, Hautcharage L-4940, Luxembourg; Environmental and Industrial Biotechnologies, Luxembourg Institute of Science and Technology, Hautcharage L-4940, Luxembourg; Environmental and Industrial Biotechnologies, Luxembourg Institute of Science and Technology, Hautcharage L-4940, Luxembourg; The Faculty of Science, Technology and Medicine, University of Luxembourg, Esch-sur-Alzette L-4365, Luxembourg; Environmental and Industrial Biotechnologies, Luxembourg Institute of Science and Technology, Hautcharage L-4940, Luxembourg; Environmental and Industrial Biotechnologies, Luxembourg Institute of Science and Technology, Hautcharage L-4940, Luxembourg; Environmental and Industrial Biotechnologies, Luxembourg Institute of Science and Technology, Hautcharage L-4940, Luxembourg; Environmental and Industrial Biotechnologies, Luxembourg Institute of Science and Technology, Hautcharage L-4940, Luxembourg; Environmental and Industrial Biotechnologies, Luxembourg Institute of Science and Technology, Hautcharage L-4940, Luxembourg; Faculty of Energy and Environment, Northumbria University, Newcastle-upon-Tyne NE1 8QH, United Kingdom; Department of Molecular Sciences, Swedish University of Agricultural Sciences, BioCentre, Uppsala SE-75007, Sweden; Department of Molecular Sciences, Swedish University of Agricultural Sciences, BioCentre, Uppsala SE-75007, Sweden; Department of Molecular Sciences, Swedish University of Agricultural Sciences, BioCentre, Uppsala SE-75007, Sweden; Department of Biomedical and Clinical Sciences, The Division of Cell and Neurobiology, Linkoping University, Linkoping 581 83, Sweden; Department of Molecular Sciences, Swedish University of Agricultural Sciences, BioCentre, Uppsala SE-75007, Sweden; The Faculty of Science, Technology and Medicine, University of Luxembourg, Esch-sur-Alzette L-4365, Luxembourg; Luxembourg Centre for Systems Biomedicine, University of Luxembourg, Esch-sur-Alzette L-4365, Luxembourg; Environmental and Industrial Biotechnologies, Luxembourg Institute of Science and Technology, Hautcharage L-4940, Luxembourg; Rectorate, Université du Luxembourg, Maison du Savoir, Esch-sur-Alzette L-4365, Luxembourg; Environmental and Industrial Biotechnologies, Luxembourg Institute of Science and Technology, Hautcharage L-4940, Luxembourg

**Keywords:** anaerobic digestion, genomics, microbial isolation, syntrophic propionate oxidation

## Abstract

The candidate phylum Cloacimonadota is frequently detected in anoxic environments such as anaerobic digestion (AD) reactors, hydrothermal vents, and deep-sea sediments, yet its metabolism remains poorly understood. Metagenomic evidence suggests capacities for amino acid fermentation, carbohydrate degradation, as well as a potential role in syntrophic propionate oxidation (SPO), a key bottleneck in AD. However, a complete methylmalonyl-CoA (mmc) pathway, central to SPO, has not been previously identified in Cloacimonadota genomes. Here, we report results from an acidified lab-scale anaerobic baffled reactor fed with sugar beet pulp, where an increase in the relative abundance of Cloacimonadota correlated with recovery of methanogenesis, resulting in increased methane content in the produced biogas. Metagenomic and metatranscriptomic analyses enabled metabolic reconstruction of the dominant Cloacimonadota operational taxonomic unit (OTU). Furthermore, using a curated database of 204 genome-resolved Cloacimonadota species, we characterized the phylum-level metabolic potential. Comparative genomics revealed alternative proteins, including 2-oxoglutarate:ferredoxin oxidoreductase and aspartate aminotransferase, likely to substitute for missing enzymes in the classical mmc pathway. These proteins were widely distributed and highly conserved across the analyzed Cloacimonadota genomes, suggesting that this variant of the SPO pathway could represent a phylum-specific trait. Moreover, we hypothesize that these alternative pathway steps may link propionate metabolism to protein degradation and poly-γ-glutamate biosynthesis. Network analysis identified the methanogenic archaeon *Methanothrix* as a potential syntrophic partner, an interaction further supported by propionate-fed enrichment cultures showing co-occurrence of Cloacimonadota and *Methanothrix* species. Our study sheds light on the Cloacimonadota metabolism, advancing our understanding of their ecological roles and potential for biotechnological applications.

## Introduction

The candidate phylum Cloacimonadota, formerly *Candidatus* Cloacimonetes, is a part of the *Fibrobacteres–Chlorobi–Bacteroidetes* superphylum. Cloacimonadota comprises Gram-negative bacteria that are metabolically poorly characterized. Despite several isolation attempts, no pure cultures have been obtained, although successful enrichments have been reported [1, 2]. Cloacimonadota are predominantly found in anoxic environments, often associated with methanogenic habitats such as anaerobic digestion (AD) reactors, deep-sea sediments, and hydrothermal vents [3–5]. In full-scale AD units, they typically constitute between 1% and 12% of the bacterial community [3]. In deep-sea anoxic waters, Cloacimonadota accounts for between 5% and 15% of bacterial 16S rRNA gene reads [4].

The first genome sequence of Ca. Cloacimonas acidaminovorans was reconstructed over 15 years ago from a metagenomic (MG) library of a municipal wastewater treatment plant (WWTP), where it represented 2% of a fosmid library [1]. Since then, multiple draft genomes of Cloacimonadota have been recovered from lab-scale and full-scale AD reactors [2, 6, 7], hydrothermal vents [4], animal gut-associated habitats [8, 9], among others [5]. Based on the encoded metabolic potential, Cloacimonadota are recognized as amino acid-fermenting bacteria [1, 2]. They encode a diverse repertoire of carbohydrate-active enzymes [5], and have been reported to be associated with cellulose degradation [10]. However, the main interest in this uncultured bacterial phylum stems from its presumed role in syntrophic propionate oxidation (SPO), a key process that helps overcome one of the major bottlenecks to efficient methane production in AD reactors [11]. Several studies have linked increased abundance of different Cloacimonadota species to propionate consumption [7, 12], suggesting that SPO might be a common feature of the phylum. Conversely, decreases in their relative abundance have also been reported under conditions of elevated propionate levels [13]. No reconstructed Cloacimonadota genome contains a complete set of genes encoding the key enzymes of the methylmalonyl-CoA (mmc) pathway, which is widely recognized as being involved in SPO. Furthermore, the lack of cultured representatives complicates the elucidation of their role in the SPO process and currently limits the group to *Candidatus* status only.

This study was motivated by the enrichment of an uncultured Cloacimonadota OTU in a lab-scale acidified AD system, which correlated with propionate consumption and increased methane production. To explore its metabolic potential for SPO, we reconstructed its complete genome and compiled a comprehensive Cloacimonadota genome database to further clarify the ecological role of this phylum in AD. Comparative genomic analyses revealed a putative mmc pathway, with alternative proteins potentially compensating for missing steps of the classical mmc pathway and suggested that propionate oxidation may be linked to protein degradation and poly-γ-glutamate (PGA) production in Cloacimonadota. The widespread presence of this pathway across nearly all Cloacimonadota genomes further indicates that SPO may be a phylum-wide trait. Correlation network analysis (CNA) also highlighted potential syntrophic interactions with methanogenic archaea of the genus *Methanothrix* and alternatively *Methanosarcina*.

## Material and methods

### Anaerobic baffled reactors: operation, sampling, and 16S rRNA gene amplicon sequencing

**Figure 1:**
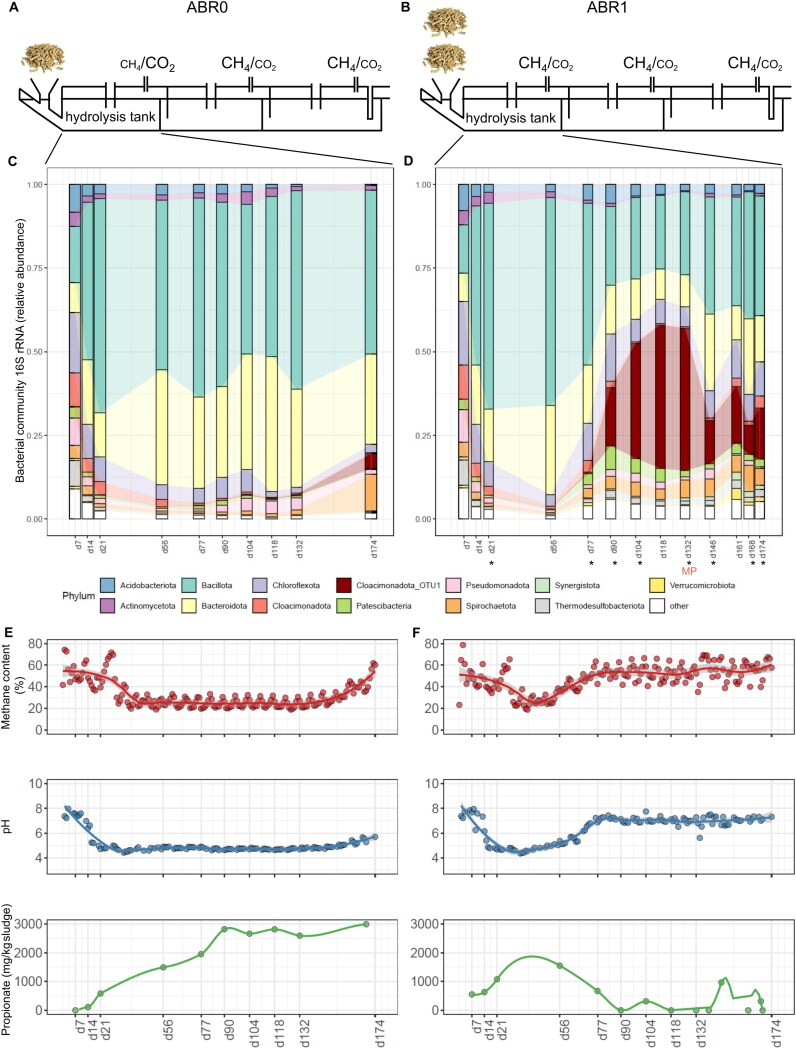
Overview of the ABR. Experimental design and operation (A and B), the microbial communities established in the hydrolysis tanks (C and D), and the recorded parameters (E and F) for the ABR0 and ABR1 reactors (left and right panel, respectively). The abundance of Cloacimonadota OTU_1 is highlighted in the graphs. Samples analyzed for MG and MT are marked with an asterisk and metaproteomics are indicated with MP.

Four three-compartment anaerobic baffled reactors (ABR0-3) were operated under mesophilic conditions for 174 days, with ABR2 and ABR3 specifically dedicated to the bioaugmentation trials. All reactors were inoculated with anoxic sludge sourced from a full-scale methanogenic digester treating activated wastewater sludge, and samples were regularly collected from each ABR compartment. Further details are elaborated in the Supplementary Material (Methods 1.1). DNA and RNA macromolecules were co-extracted on the days indicated in Fig. 1, using the AllPrep DNA/RNA Mini Kit (Qiagen, Hilden, Germany) and following the manufacturer’s protocol. Bacterial and archaeal 16S rRNA gene amplicon libraries were prepared using a previously optimized Illumina-compatible sequencing approach [3]. CNA was conducted on normalized 16S rRNA gene reads following the previously developed method [14] with further modifications. The complete protocols are described in Supplementary Material (Methods 1.2), and additional details are provided in Supplementary Dataset 1 (Tables S1–S4).

### Metagenomics, metatranscriptomics, and metaproteomics

Samples collected at eight time points from the first compartment (hydrolysis tank) of ABR1 were selected for MG sequencing (Fig. 1D). Sequencing libraries were prepared with the Nextera XT kit (Illumina) and sequenced using the NextSeq 500 system (Illumina; University of Luxembourg), generating 49.9 Gb of 150 bp MG paired-reads. Omics samples were analyzed at time points corresponding to the occurrence of OTU_1 at high relative abundance. One sample (Day 132) was additionally sequenced using long-read Nanopore approach (BaseClear, The Netherlands) producing 11 Gb. Short Illumina reads were quality trimmed in CLC Genomics Workbench v23.0.5 (Qiagen), using a phred quality score of 20, minimum length of 50 and allowing no ambiguous nucleotides. Quality-trimmed reads were assembled using the CLC’s *de novo* assembly algorithm in a mapping mode, using automatic bubble size and word size, minimum contig length of 1000, mismatch cost of 2, insertion cost of 3, deletion cost of 3, length fraction of 0.9, and similarity fraction of 0.95. Metatranscriptomics (MT) was conducted on the same eight samples as MG (Fig. 1D), generating 82.1 Gb of 150 bp paired-reads. Library preparations and data analysis followed previously optimized in-house protocols [15]. Canu-1.8 was used to process Nanopore reads and generate the assembly [16]. Illumina MG reads were mapped back to the Nanopore assembly, and binning and bin refinement, including re-assembly, were performed using MetaWRAP v1.3 [17], which combined binning results of CONCOCT [18], MaxBin2 [19], and Metabat2 [20]. In total, 52 refined metagenome assembled genomes (MAGs) were retained with >50% checkM [21] completeness and below 10% contamination (Supplementary Dataset 2, Table S5). Bin.36, corresponding to Cloacimonadota OTU_1, was re-assembled by extracting mapping Illumina and Nanopore reads as input for UniCycler [22], which resulted in a circular genome of 2.3 Mb. Functional and taxonomic annotation was performed for the reconstructed MAGs (see below). llumina MG and MT reads (following rRNA removal) were mapped back to the final MAG set with bwa-mem v0.7.12 [23] and Nanopore reads were mapped with minimap2 v2.17-r941 [24]. Coverage and TPM values on MAG level were determined with coverM v0.7 [25]. For MT analysis, salmon v1.4 [26] was used to quantify the transcriptional abundance of predicted genes (including genes from MAGs and unbinned contigs) including sequence bias correction and the –meta option. Metaproteomics (MP) was applied to two samples in duplicates corresponding to the time point Days 118 and 132 (Fig. 1) to confirm the presence of enzymes from the mmc pathway of Cloacimonadota origin. Further details on the methods used in this study are described in Supplementary Material (Methods 1.3). Results corresponding to these methods are available in Supplementary Datasets 2, 3, and  4.

### Collection of Cloacimonadota genomes and phylogenetic analysis

The Cloacimonadota genome database was constructed by integrating Cloacimonadota-annotated genomes downloaded from NCBI in July 2025, genomes reconstructed in this study, and others [27] (Supplementary Dataset 2, Tables S6–S7). Additionally, taxonomy assignment was performed using GTDB-Tk v2.6.1 and database release 226 [28], resulting in 558 retained genomes, meeting the criteria for medium to high quality MAGs (e.g. completeness higher than 50% and contamination lower than 10%). All genomes underwent dereplication using dRep v3.6.2 [29] with “gANI -sa 0.965 -nc 0.6”. Genome dereplication at the estimated species level resulted in the formation of 204 genome clusters (GC). Average Nucleotide Identity (ANI) and Average Aminoacid Identity (AAI) were calculated for enriched GS using OrthoANI [30] and the enveomics suite [31], respectively. Following the microbial species delineation criteria previously proposed [32], we considered two nearly complete MAGs with an average ANI below 96.5% over at least 60% of the aligned genome to represent distinct microbial species. Upon cultivation of representative strains, members of these GC are likely to represent novel species. A whole-genome phylogenetic tree was constructed using Phylophlan3 v.3.1.68 [33] and visualized with iTOL [34].

### Gene calling, functional annotation, protein cluster generation, and comparative genomic analysis

Gene calling was executed using Prodigal [35] within Prokka v1.14.6 [36]. All predicted proteins underwent functional annotation (Supplementary Dataset 2, Tables S8-S10), including ortholog searches with emapper-2.0.1 and utilization of the eggNOG 5.0 database [37]. Proteins were grouped into protein clusters (PCs) using MMseqs2 [38] with the parameters: “--min-seq-id 0.3 -c 0.8 --cov-mode 1”. The functional assignment to the Kyoto Encyclopedia of Genes and Genomes (KEGG) orthologous categories (KOs) was done with GhostKoala [39]. PCs were functionally annotated using both emapper and GhostKoala. Enrichment of KOs was performed with Maaslin2 v1.22.0 [40] in R.4.5 with analysis method “LM” and correction “BH” and no normalization or transformation as KO counts per assigned habitat (Supplementary Dataset 2, Table S8) were compared. To characterize the metabolic potential of Cloacimonadota versus other major phyla present in the AD reactors, a previously generated database of MAGs was used [41]. The KEGG database was used to assign gene functionalities to the MAGs. Thermodynamics calculations of enzymatic reactions were made as previously described [42]. Gibbs free energy of formation values for the various SPO steps were obtained from previous studied [43–45].

### Cloacimonadota isolation trials and genome reconstruction of enriched species

Due to the time lag between sample collection, data acquisition, and analysis, the isolation trials were conducted after the ABR experiment had terminated. Therefore, we used the same seeding sludge as the ABR inoculum for the isolation experiment, rather than the reactor-enriched sludge. Given that PGA is a known virulence factor in other species and can confer nonspecific antibiotic resistance, sludge inoculum was incubated with broad-spectrum antibiotics (ampicillin, streptomycin, vancomycin, and ciprofloxacin) at the time of media inoculation, using modified DSM 621 and other custom media with various carbon sources. Enrichments were maintained under anoxic conditions, in closed serum bottles for three months at either 37°C, or room temperature with intermittent mixing. Further isolation efforts were designed to specifically target enriched Cloacimonadota. For a detailed description of the enrichment study design, see Supplementary Material (Methods 1.4). To monitor the enrichment of Cloacimonadota, the enrichment cultures were periodically analyzed using Nanopore-based 16S rRNA gene sequencing. These 16S rRNA gene data are not included in the manuscript, as they were used solely to monitor the presence or absence of target taxa. One culture, initially amended with D-glucose, significantly enriched in Cloacimonadota and was subsequently passaged on modified DSM 621 medium supplemented with propionate (10 g.L^−1^) as the main carbon source and incubated at room temperature with gentle mixing (80 rpm).

To enable genome reconstruction and further characterization of the species enriched in the examined propionate-fed culture, sequencing was performed on the MiSeq system (Illumina) using the v3 reagent kit (2 × 250 bp paired-end reads), with library preparation carried out using the Illumina DNA Prep kit, and the bioinformatic analysis was performed as described above.

### Fluorescence *in situ* hybridization and microscopy

**Figure 2:**
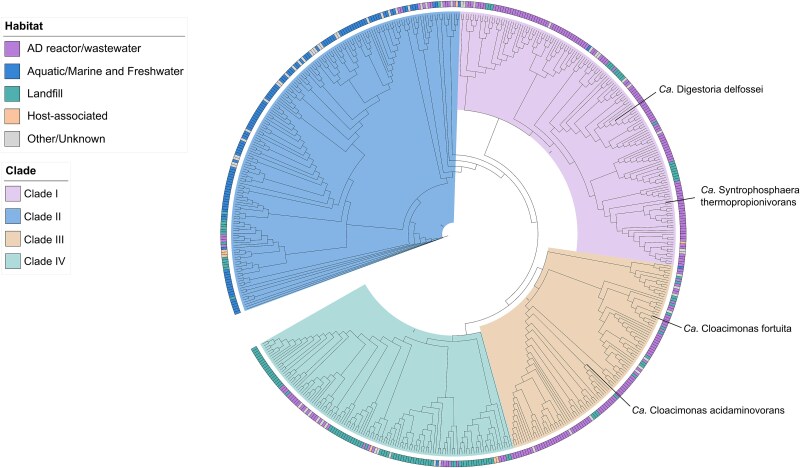
Phylogenetic placement of *Cloacimonadota* genomes based on a maximum-likelihood tree constructed from concatenated conserved protein alignments. The positions of the two candidate species identified in this study are highlighted. Four major tree clades are color-coded according to the legend, and the outer circle indicates the habitat origin of each genome.

Fluorescence *in situ* hybridization (FISH) was employed to assess the laboratory culture enriched on propionate (10 g.L^−1^), initially confirming a significant enrichment of Cloacimonadota as indicated by 16S rRNA gene amplicon sequencing and MGs. To specifically visualize the presence of Cloacimonadota in this culture, we designed probes CloaPFishV1 (5′ GCACCATGATGTTGCG 3′) and CloaOFishV2 (5′ GCGCCATGATGTTGCG 3′), which were utilized in equimolar ratio. These probes, differing by a single nucleotide (underlined), were specifically tailored to target most of the Cloacimonadota species within the tree clades I and III (Fig. 2). The design of these probes relied on aligned partial 16S rRNA gene sequences of Cloacimonadota from the previous study [3], and their validation was conducted using the FISH probe search tool implemented in SILVA (https://www.arb-silva.de/search/testprobe/). The protocol is outlined in Supplementary Material (Methods 1.5). In addition, DAPI staining was used to label bacterial DNA and to visualize the PGA surrounding the Cloacimonadota flocs, following the previously described method [46], which demonstrated that DAPI can be used for *in situ* detection of PGA. Gram-staining protocol was applied to the PBS-washed culture and observed under light microscopy (Zeiss).

## Results and discussion

### Novel Cloacimonadota OTU_1 enriched in reactors operated with high organic loading rates

Four three-compartment ABR reactors (ABR0–ABR3) fed with SBP were used in this study. Although they were originally designed to assess the effect of physically separating AD stages on methane production, this work focuses on the first compartment only, the hydrolysis tank (Fig. 1). Results from the other compartments are provided in the Supplementary Dataset 1 (Tables S1–S4) and Supplementary Material (Figs S1–S4). ABR0 was operated at a moderately increasing but overall low OLR, whereas ABR1 was subjected to a substantially higher OLR. Due to the highly fermentable nature of SBP, the hydrolysis tank became acidified early in the experiment for both reactors, regardless of the applied OLR. The relative abundance of Cloacimonadota (OTU_1) in the hydrolysis tank of ABR1 increased from close to 0% at Day 56 and 0.5% at day 77 to 43% at day 132. This increase was negatively correlated with measured propionate concentrations (ρ −0.97, *P* < .05; Fig. 1). The gradual decline in propionate concentration ranged from 1550 mg. kgsludge^−1^ at Day 56 to 670 mg. kgsludge^−1^ at Day 77 to 0 mg. kgsludge^−1^ at Day 90. The reduction in VFAs led to a rise in pH (Supplementary Dataset 1, Table S1), restoring neutral conditions and resulting in increased methane content in the biogas in the initially acidified hydrolysis tank of ABR1 (Fig. 1B, D, F). In contrast, the same compartment of ABR0 did not enrich Cloacimonadota and functioned as a typical hydrolysis tank, with pH oscillating around acidic values (pH 5; Fig. 1A, C, E). Whereas these findings suggest that Cloacimonadota OTU_1 may contribute to SPO, consistent with observations in other Cloacimonadota representatives [11], the increasing abundance of several other OTUs taxonomically assigned to *Thermotogota* (ρ −0.72), *Fibrobacterota* (ρ −0.72), *Patescibacteria* (−0.69), and *Armatimonadota* (ρ −0.68) also negatively correlated with propionate concentrations (*P* < .005). Nevertheless, based on taxonomic annotation, none of these OTUs are known to be associated with SPO, and their relative abundance at the phylum level was much lower (<1%) compared to OTU_1 (maximum 43%). Consequently, they were not analyzed further in this study.

To assess the potential of Cloacimonadota to counteract propionate accumulation, we conducted a bioaugmentation experiment using ABR2. Its hydrolysis tank, also fed with high OLR of SBP, had become acidified and ceased methane production. To restore its performance, we supplemented it with sludge enriched in the specific Cloacimonadota OTU_1, from the hydrolysis tank of the ABR1. After five rounds of bioaugmentation, the acidified compartment resumed methane production, and the pH bounced back to neutral values (Supplementary Material, Results and discussion 2.1; Fig. S5). Concurrently, Cloacimonadota OTU_1 established itself in the hydrolysis tank. As such, we concluded that AD sludge enriched in Cloacimonadota OTU_1 can be considered an effective bioaugmentation agent for restoring methane production in acidified AD reactors, as previously recognized in a patent publication [47].

### Genome reconstruction and metabolic pathway analysis of *Candidatus* Digestoria delfossei: a potential syntrophic propionate oxidation bacterium

Using a combination of short- and long-read sequencing approaches, we reconstructed genomes of 52 microbes residing in the hydrolytic tank of ABR1, comprising eight archaeal and 44 bacterial genomes of medium to high draft genome quality (according to MIMAG standards [48]), mainly representing *Bacteroidota, Spirochaetota, Patescibacteria, Planctomycetota, Desulfobacterota*, and *Halobacterota* phyla (Supplementary Dataset 2, Table S5; Supplementary Material, Figs S6 and S7). Genome reconstruction of Cloacimonadota OTU_1 yielded a complete, closed genome assembly. It is 2.3 Mbp long, with a GC content of 54.3%, and it contains two rRNA operons (Supplementary Dataset 2, Table S6). The genome was compared to the two previously described species of Cloacimonadota, Ca. Cloacimonas acidaminovorans [1] and Ca. Syntrophosphaera thermopropionivorans [7] to assess its taxonomic placement. Based on ANI and AAI thresholds for genus and species delineation, it falls below the accepted cutoffs (ANI < 96.5, AAI 60%–80%), indicating it represents a new lineage. Adhering to the rules of the Code of Nomenclature of Prokaryotes Described from Sequence Data (SeqCode) for high-quality taxonomic description of uncultivated taxa [49], we propose naming it *Candidatus* Digestoria delfossei gen. nov., sp. nov. (Supplementary Material, Results and discussion 2.8). Based on genome reconstruction, the Ca. Digestoria delfossei encodes a complete glycolysis pathway with pyruvate orthophosphate dikinase exchanging for pyruvate kinase, as previously shown in Cloacimonadota genomes [5], and it has capacity to oxidize pyruvate further to acetyl-CoA and acetate. It encodes the metabolic potential for beta-oxidation and protein degradation (i.e. encoding multiple peptidases, including aspartic, cysteine, metallo-, and serine peptidases) as well as a complete pathway for the conversion of histidine to glutamate. Although Cloacimonadota have been suggested to represent SPO bacteria (SPOB), a complete mmc pathway for propionate oxidation was not found in the genome (Supplementary Material, Results and discussion 2.3), with the same missing steps as previously reported for this phylum [11].

### Identifying potential syntrophic partners of Ca. Digestoria delfossei

To investigate which methanogen(s) could represent potential syntrophic partner(s) of Ca. Digestoria delfossei (OTU_1), we performed a CNA based on a 16S rRNA gene amplicon dataset comprising nearly 300 samples from both the present and previous study [50]. From this network, *Methanothrix sp.* showed the strongest co-occurrence signal, suggesting it could serve as the primary archaeal syntrophic partner for Ca. Digestoria delfossei (Supplementary Dataset 1, Table S4). Although *Methanothrix* sp. is traditionally classified as an obligate aceticlastic methanogen, genomic analyses have revealed the presence of a complete gene set associated with hydrogenotrophic methanogenesis [51]. Gene expression profiles indicated that genes from both aceticlastic and hydrogenotrophic pathways were expressed at different stages of ABR operation, and MP analyses further detected peptides corresponding to enzymes involved in both methanogenic routes (Supplementary Dataset 3, Tables S11–S14). Nevertheless, previous culture-based experiments provided no evidence that *Methanothrix* could solely rely on hydrogenotrophic methanogenesis [51]. We therefore tentatively hypothesize that *Methanothrix* could instead participate in direct interspecies electron transfer (DIET) with members of the Cloacimonadota phylum in AD reactors. DIET has previously been suggested for Cloacimonadota [11], and we further identified genes encoding type IV pilus assembly proteins in almost all AD Cloacimonadota genomes. In Ca. Digestoria delfossei, the entire pilus operon was identified and is encoded directly upstream of several mmc pathway genes (i.e. the cluster containing mmc epimerase and 2-oxoglutarate oxidoreductase genes), indicating potential co-regulation. Pilus-related genes were expressed throughout the experiment, consistent with a role in interspecies interactions (Supplementary Dataset 3, Table S13). DIET between *Methanothrix* and *Geobacter* species has previously been experimentally demonstrated [52], tentatively supporting the capacity of *Methanothrix* to exchange electrons with Cloacimonadota via a similar mechanism. Conversely, direct uptake of acetate by *Methanothrix* produced during SPO can also take place, and both hypotheses require further validation.

Other potential syntrophic archaeal partners of Ca. Digestoria delfossei were taxonomically affiliated with *Methanosarcina* sp. *and Methanospirillum* sp. (Supplementary Material, Results and discussion 2.2; Supplementary Dataset 1, Table S4). Both could potentially participate in syntrophic interactions via H₂, whereas *Methanosarcina* can additionally metabolize acetate to methane.

### Characterization of encoded metabolic potential of Cloacimonadota

**Figure 3:**
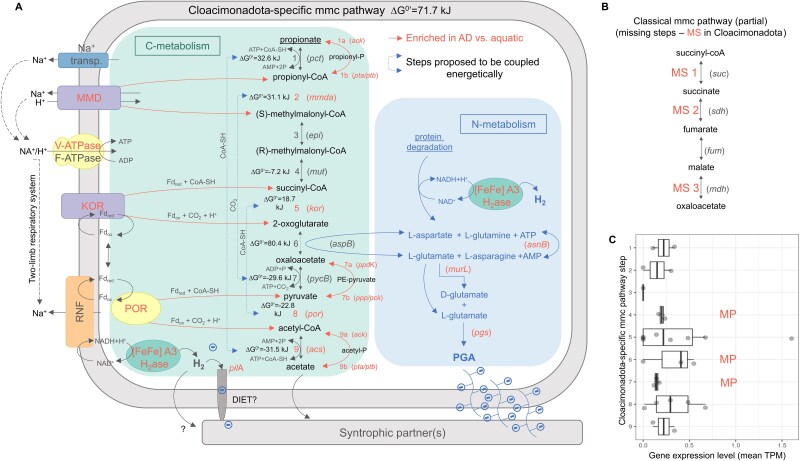
A simplified representation of the alternative propionate oxidation pathway proposed for Cloacimonadota (from AD systems), highlighting its potential links to nitrogen metabolism and PGA production (A). Missing steps (MS) compared to the typical mmc pathway (B). Genes and reaction steps overrepresented in AD-associated Cloacimonadota genomes, compared to those from aquatic habitats, are highlighted in orange. Dashed lines indicate hypothetically energy coupled reactions. MT abundances of genes involved in different pathway steps (C). Corresponding proteins identified in the MP analysis are indicated with the letter “MP”. Additional details can be found in Supplementary dataset 3, Tables S11–S14. MMD—methylmalonyl-CoA decarboxylase, KOR—2-oxoglutarate: ferredoxin oxidoreductase, POR—pyruvate ferredoxin oxidoreductase, RNF—Rhodobacter nitrogen fixation, [FeFe] A3 H_2_ase—hydrogenase of group A3, DIET—direct interspecies electron transfer.

As Ca. Digestoria delfossei is the third member of the Cloacimonadota suggested to perform SPO in AD systems, we hypothesized that, unlike currently known SPOB, which are scattered across multiple bacterial phyla [11], SPO may represent a phylum-wide trait within the Cloacimonadota. To test this hypothesis and to further evaluate the ecological function of this candidate phylum across different environments, we assembled a collection of Cloacimonadota genomes. It included MAGs reconstructed in this and our previous studies, and additional MAGs obtained from public databases, summing up to 558 genomes, further representing 204 GC (GC1–GC204; Supplementary Dataset 2, Tables S5–S10). A phylogenetic reconstruction based on concatenated protein comparisons revealed a grouping of Cloacimonadota into four major clades (I, II, III, IV), which largely reflected their environmental origin, representing AD reactors including wastewater systems (mainly clades I and III), aquatic (marine and freshwater; clade II), and landfill (clade IV; Fig. 2). Functional annotation identified 2881 distinct KOs, covering an average of 44.6% ± 4.9% of coding sequences (mean 1733) per MAG (Supplementary Dataset 2, Tables S8–S10). Among the functionally assigned genes, clade II representing mainly aquatic environments, showed the highest KO diversity (2534 different categories assigned, 811 of those appearing only in clade II), suggesting broader functional potential than in AD and landfill-associated lineages (average 1454 ± 78 KOs for clades I, III, and IV). A comparison between AD and aquatic, the two environments with the highest number of Cloacimonadota MAGs retrieved in our analysis, indicated 371 KOs enriched in AD MAGs (coef <0, *q*-value <0.05) and 190 in aquatic MAGs (coef >0, *q*-value <0.05; Supplementary Dataset 2, Table S8). Genes enriched in AD genomes were dominated by core information-processing functions (replication, transcription, and translation), cell envelope biogenesis, and widely distributed metabolic pathways. Among them, 230 corresponded to enzymes, including diverse peptidases (aspartic, cysteine, serine, and metallopeptidases) as well as other amino acid-related enzymes (e.g. aminotransferases and multiple aminoacyl-tRNA synthetases), suggesting a preference for amino acid metabolism. Among transporters, several phosphate transport systems were identified, but saccharide ABC transporters were predominant, further indicating a potential preference for carbohydrate utilization. Enrichment of genes encoding flagellar assembly proteins and pilus systems in AD genomes also suggests an increased capacity for bacterial motility compared to aquatic Cloacimonadota. Differences in the enrichment of several mmc pathway genes were observed between aquatic and AD MAGs, with some genes missing or present in altered forms in aquatic MAGs (Fig. 3A; Supplementary Dataset 2, Table S8). Several mmc pathway genes, including acetyl transferase, methylmalonyl decarboxylase, methylmalonyl epimerase, oxoglutarate:ferredoxin oxidoreductase, malate dehydrogenase, and succinyl-CoA synthetase, were co-encoded in the same genomic location in aquatic Cloacimonadota, forming a cluster akin to the mmc cluster in other known SPOB [11, 53].

### Proposal for an alternative syntrophic propionate oxidation pathway in Cloacimonadota

As previously reported [11], analysis of two representative Cloacimonadota genomes from methanogenic systems revealed three missing steps of the classical mmc pathway in this phylum (Fig. 3A and B). These include the ATP-generating conversion of succinyl-CoA to succinate catalysed by succinyl-CoA synthetase (missing step 1), succinate oxidation to fumarate catalysed by succinate dehydrogenase (missing step 2), and malate oxidation to oxaloacetate catalysed by malate dehydrogenase (missing step 3). Previous phylum-level analysis of Cloacimonadota genomes also provided no evidence for genes encoding propionate-CoA transferase [5], a key enzyme in the conversion of propionate to propionyl-CoA (step 1, Fig. 3A). To explore alternatives to the missing mmc pathway steps in AD Cloacimonadota, we first manually screened the genome of Ca. Digestoria delfossei for genes encoding proteins that could potentially substitute for the missing mmc pathway steps. We then assessed the presence of these alternative mmc pathway genes across other Cloacimonadota genomes. Based on our analysis, we hypothesize that in Cloacimonadota, the missing steps 1–3 of the mmc pathway (Fig. 3B) are substituted first by succinyl-CoA dehydrogenation to 2-oxoglutarate catalysed by a transmembrane enzymatic complex, 2-oxoglutarate ferredoxin oxidoreductase (kor; Fig. 3A; Supplementary Dataset 3, Tables S11 and S12). Subsequently, the generated 2-oxoglutarate is converted by aspartate aminotransferase (aspB) to oxaloacetate, which is further metabolized to acetate via the classical mmc reaction steps. Aspartate aminotransferase facilitates the reversible interconversion of L-aspartate and α-ketoglutarate with oxaloacetate and L-glutamate through a ping-pong catalytic cycle [54], putatively linking carbohydrate and protein metabolisms in Cloacimonadota.

We further screened the other MAGs generated in this study from the ABR reactor to determine whether this alternative mmc pathway might also be present in other species and found that several *Bacteroidota* MAGs contained at least one gene encoding an enzyme associated with each step of the pathway (Supplementary Material, Results and Discussion 2.3 and Figs S8–S9). This observation is somehow consistent with previous studies reporting the potential for SPO in diverse bacterial lineages [55]. Conversely, it may also indicate a species’ capacity for propionate production. A comprehensive characterization of the alternative mmc pathway in other species, however, is beyond the scope of the present study.

### Comparison of the alternative syntrophic propionate oxidation route with the classical mmc pathway

The thermodynamic calculation of the alternative mmc pathway (DG^0^’ = 71.7 kJ; Fig. 3) corroborated the energetic agreement with the classical mmc pathway (DG^0^’ = 73 kJ) [56]. Like the classical mmc pathway, the energy-dependent steps in Cloacimonadota may be coupled with energy-yielding reactions, supported by coupled reactions thermodynamics (Fig. 3A). Cloacimonadota have the potential to connect the initial endergonic step of propionate activation to the last exergonic step of acetyl-CoA deactivation (steps 1 and 9), which is also found in several other SPOB [11]. Whereas phosphate butyryltransferase (K00634) seems prevalent in Cloacimonadota genomes (Supplementary Dataset 2, Table S8), butyrate oxidation has not been reported by members in this phylum so far. This suggests that this enzyme could have been misidentified as a phosphate propionyl transferase (encoded by the *pta* gene; Fig. 3A). Indeed, information from the Brenda database suggests that the same enzyme can accept propionyl-CoA alongside butyryl-CoA in Listeria monocytogenes [57]. Step 2 entails a membrane-bound Na^+^—transporting methylmalonyl decarboxylase (*mmd)* catalysing carboxylation of propionyl-CoA to mmc, potentially coupled to the energy-yielding oxaloacetate decarboxylation to pyruvate (step 7). Methylmalonyl decarboxylase relies strictly on Na^+^ ions for activity. Transportation across the cytoplasmic membrane creates a sodium ion motive force, utilized for ATP synthesis. This enzyme, along with the putative phosphate propionyltransferase (*pta*, step 1) and mmc epimerase (*epi*, step 3) are among the most frequently encountered PCs in the Cloacimonadota genomes (Supplementary Dataset 2, Table S9). Their high conservancy at the protein level suggests that they play a fundamental role in the metabolism of this phylum. The transmembrane 2-oxoglutarate:ferredoxin oxidoreductase (*kor*) is encoded next to the mmc epimerase, likely forming a single operon. The reaction step 5 is likely thermodynamically coupled to one catalysed by pyruvate:ferredoxin oxidoreductase (“por”, step 8). In the apparent absence of a ferredoxin-dependent hydrogenase in Cloacimonadota, previously suggested to rely solely on an NADH-dependent enzyme [58], KOR may recycle electrons from reduced ferredoxin, which in other species would typically be used for H₂ production [59]. The conversion of 2-oxoglutarate to oxaloacetate is putatively linked to the transamination of L-aspartate and L-glutamate (step 6). This reaction likely connects carbohydrate and protein metabolisms, with 2-oxoglutarate acting as a major carbon skeleton to assimilate nitrogen from protein degradation. Indeed, Cloacimonadota have previously been reported to ferment amino acids to 2-oxoacids [1, 7]. The presence of genes encoding enzymes involved in the interconversion/transformation of glutamate ↔ glutamine (i.e. glutamate dehydrogenase, glutamine synthase) and aspartate ↔ asparagine (asparaginase) in nearly all AD Cloacimonadota genomes, further suggests a potential for efficient integration of carbon and nitrogen metabolism via 2-oxoacids.

RNA sequencing (MT) revealed the transcription of all genes identified as part of the proposed alternative mmc pathway (Supplementary Dataset 3, Table S13), except for mmc epimerase (*epi*, step 3; Fig. 3C). However, the read counts for these genes were relatively low in our MT dataset, which was dominated by transcripts originating from methanogenic archaea (Supplementary Material, Fig. S7). MP analysis further identified several proteins representing various steps of the proposed pathway under high propionate conditions, including PTA (step 1b), MUT (step 4), ASPB (step 6), PYC B (step 7), as well as components of RNF, trimeric A3 [FeFe] hydrogenase, and V-ATPase complexes (Fig. 3; Supplementary Dataset 3, Table S14). Additionally, glutamate dehydrogenase and L-asparaginase were abundant, indicating active pathways involved in nitrogen metabolism. Collectively, these findings provide tentative support for the validity of the newly proposed alternative mmc pathway in Cloacimonadota.

### Unique phylum-wide capacity for syntrophic propionate oxidation in Cloacimonadota within anaerobic digestion communities

**Figure 4:**
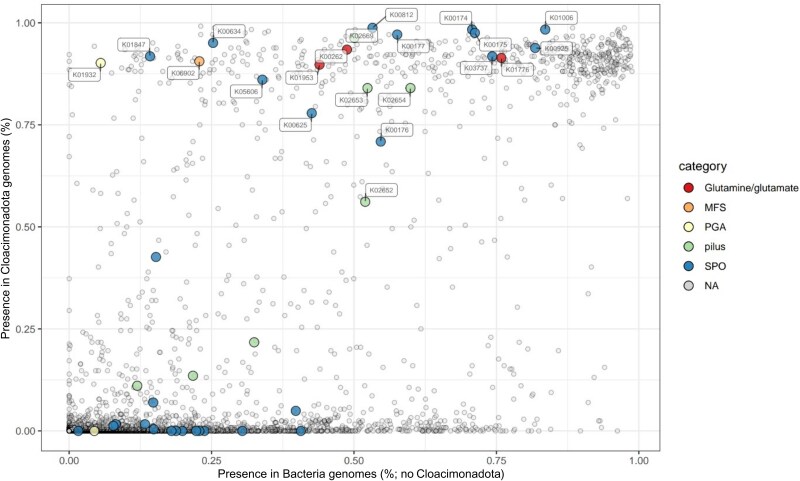
Visual representation of KEGG Orthologies (KOs) in AD Cloacimonadota genomes compared to other AD bacteria. KOs involved in syntrophic propionate degradation and associated pathways (referenced in Fig. 3) are highlighted in the graph. MFS—Major facilitator superfamily transporter, PGA—poly-ɣ-glutamate, SPO—syntrophic propionate oxidation.

In continuation, we aimed to determine whether the phylum-wide metabolic capacity for SPO is unique to Cloacimonadota or also frequently occurs in other bacterial phyla within the AD community (Supplementary Dataset 4, Table S15). To achieve this, we utilized another extended collection of previously published AD-specific MAGs [41], which we supplemented with AD Cloacimonadota genomes from our database. Acknowledging the incompleteness of some microbial genomes, we established criteria for functions represented in at least 75% of Cloacimonadota genomes and absent in at least 75% of other bacterial genomes commonly found in AD environments. We identified 58 KOs (44 with enzymatic function) strongly enriched in AD Cloacimonadota (Supplementary Material, Results and discussion 2.7; Supplementary Dataset 4, Table S15). Many mmc genes were close or slightly below the threshold, as they were also detected in some other AD bacterial phyla (median 24% of genomes), yet they were present in over 90% of the AD Cloacimonadota genomes (Fig. 4). By homology to a recently characterized major facilitator superfamily transporter responsible for propionate tolerance in a Gram-negative bacterium Pseudomonas putida [60], we proposed a K06902 as a putative propionate transporter gene in Cloacimonadota. Its presence in over 90% of analyzed Cloacimonadota genomes, whereas only roughly 23% of other AD bacteria also encoded a respective gene, further reinforces our assumption. The L-glutamate-generating aspartate aminotransferase (K00812), which we hypothesize compensates for the absence of certain steps in the classical mmc pathway, was prevalent in 99% of the analyzed Cloacimonadota genomes, sometimes occurring in multiple copies. In contrast, only 53% of the other AD microbes encoded the respective gene(s). The near-ubiquitous presence (99%) of a PGA biosynthesis gene (K01932) in AD Cloacimonadota (Supplementary Dataset 4, Table S15), compared to its rarity in other AD bacteria (identified in <6% of genomes, mainly *Bacillota*, formerly *Firmicutes*), suggests a specific capacity for PGA production (Fig. S10, see below).

### Culture enrichment of Ca. Cloacimonas fortuita, another species of Cloacimonadota from anaerobic digesters

**Figure 5:**
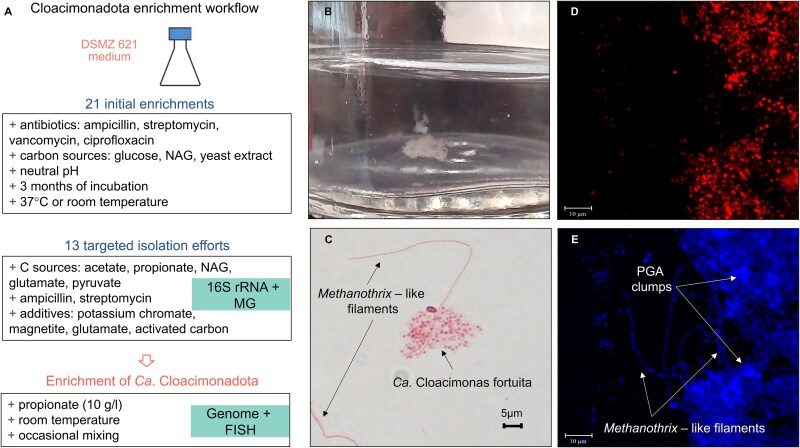
Enrichment experiment. Assay design (A) and microscopy results of cultures enriched with Ca. Cloacimonas fortuita. Formation of loosely aggregated flocs (B). Gram staining (C). FISH images showing the presence of enriched Ca. Cloacimonas fortuita in red (D). DAPI staining of the enriched culture highlighting the potential association of *Methanothrix* sp. (long filaments) with Ca. Cloacimonas fortuita and the formation of PGA clumps (E; following the PGA-DAPI staining method previously described [46]). NAG—N-acetylglucosamine, MG—metagenomics.

To validate the SPO capacity of Cloacimonadota in AD systems, we conducted a series of genome-informed cultivation trials using different anoxic media and carbon sources to selectively enrich Cloacimonadota, with the aim of isolating Ca. Digestoria delfossei (Fig. 5; Supplementary Material, Results and discussion 2.8). Initially, vancomycin was used, as a putative resistance gene was identified in its genome (data not shown). However, despite multiple attempts, we were unable to specifically enrich this species. Whereas, in ampicillin and streptomycin fortified DMS 621 medium using D-glucose as a carbon source (culture named “RT_BT_as_ph7”), we managed to enrich another Cloacimonadota species, which was subsequently passaged on a medium containing propionate as the main carbon source (Supplementary Dataset 5, Tables S16–S19). We attribute its broad non-specific antibiotic resistance to the presence of the PGA protective matrix surrounding the cells, as reported for other PGA producers [61]. The culture was rather slowly growing, forming loose white flocks (Fig. 5), which were easily destroyed if the bottle was vigorously shaken. Based on 16S rRNA gene amplicon sequencing, the corresponding OTU accounted for 56% of the community, with only two other bacterial OTUs showing significant abundance (Supplementary Dataset 5, Table S17, and Fig. S11). We discovered that the archaeon also present in the enriched culture was *Methanothrix sp.* (Supplementary Dataset 5, Table S19), initially identified as a putative partner of Ca. Digestoria delfossei based on the CNA analysis. Microscopic observation of the Gram-stained culture visualized the negatively stained sphere-shaped cells and long filaments typical of *Methanothrix* being embedded in a matrix, most probably composed of PGA (Fig. 5C). Sporadically, coccoidal aggregates typical to *Methanosarcina* genus (another putative partner based on the CNA), were also present (not shown), and fragmented genome pieces were reconstructed from MGs reads as well (Supplementary Dataset 5, Table S19). FISH analysis confirmed the presence of Cloacimonadota conglomerates, densely embedded in PGA (Fig. 5E), as could be visualized by DAPI staining of PGA [46]. The draft genome of this new Ca. Cloacimonadota species is 1.98 Mbp long, with a GC content of 36.6%, and it contains 1529 protein coding genes (Supplementary Dataset 2, Table S6). Comparing it to the two others described Cloacimonadota genomes (Ca. Cloacimonas acidaminovorans and Ca. Syntrophosphaera thermopropionivorans), shows that it represents a new species (Supplementary Material, Fig. S12) within the Cloacimonas genus, hereinafter named *Candidatus* Cloacimonas fortuita sp. nov. (Supplementary Material, Results and discussion 2.8).

### Association of poly-ɣ-glutamate production with propionate degradation in Cloacimonadota

Building on our hypothesis that SPO may be coupled with PGA formation, we examined this potential link more closely in the recovered Cloacimonadota genomes. The PGA operon of Ca. Digestoria delfossei is composed of three genes encoding proteins similar to CapB, CapC, and CapA (PgsF; Supplementary Material, Fig. S10), and exhibits the typical gene organization found in known Gram-positive PGA producers [62]. PGA production involves L-glutamic acid units derived exogenously or endogenously using 2-oxoglutaric acid as a precursor (Fig. 3A). In the absence of a complete TCA cycle in Cloacimonadota, we hypothesize that 2-oxoglutaric acid derived from the succinyl-CoA carboxylation mediated by the 2-oxoglutarate:ferredoxin oxidoreductase, could provide a carbon source for PGA synthesis, potentially linking PGA production to propionate degradation.

Overall, poly-ɣ-glutamic acid production has been primarily studied in *Bacillus* species, where the polymer was initially shown to be involved in virulence (i.e. antibiotic resistance), such as in Bacillus anthracis [61]. The presence of a capsule synthesis protein domain “IPR019079” named CapA, which is involved in PGA production, has also been observed in short chain fatty acids degrading syntrophic bacteria [63]. The physiological function of PGA in different organisms is not fully understood, and depends on whether the polymer is anchored to the peptidoglycan (i.e. a virulence factor) or released [64]. In the latter case, it can provide environmental advantages, such as helping to sequester toxic metals, decreasing salt concentration, providing a carbon source, protecting against adverse conditions, and improving biofilm formation. We speculate that in Cloacimonadota, PGA might promote syntrophic interactions by facilitating direct contact with its syntrophic partner(s), thus enhancing SPO. Moreover, highly charged polyanions such as PGA exhibit electrical conductivity behavior [65], which could potentially facilitate DIET between potential syntrophic partners.

## Conclusions

Our study highlights the ecological importance of Cloacimonadota in methanogenic environments and underlines that SPO could be a conserved functional trait within this bacterial phylum. Cloacimonadota are consistently abundant in AD reactors that exhibit propionate accumulation, suggesting a strong ecological link to propionate turnover under anoxic conditions. Genomic analysis further reveals that they encode a set of genes consistent with SPO-related metabolism, though not following the classical mmc route. Instead, they could potentially utilize an alternative mmc route, i.e. thermodynamically equivalent to the classical mmc pathway. Both, sequencing results and microscopic observations, revealed that Cloacimonadota co-occur with methanogenic archaea, suggesting potential interactions. Network analysis and Cloacimonadota enrichments point to *Methanothrix*, and alternatively *Methanosarcina* as putative archaeal partners in the syntrophic cooperation. Still, further research is needed to determine the conditions under which this partnership is established and how it functions. A key limitation of this study is the inability to isolate and maintain Cloacimonadota in pure culture. Although some species can be studied within enrichment communities, their growth can be unstable as observed here, highlighting the need for further advancements in cultivation methods. As such, future efforts should aim at isolating Cloacimonadota representatives in defined co-culture with their syntrophic partners to experimentally validate their metabolic interactions. Furthermore, elucidating the regulatory mechanisms governing the SPO and PGA-based biofilm formation may offer deeper insights into the ecological success of Cloacimonadota in the propionate-intoxicated methanogenic environments.

## Supplementary Material

wrag055_Supplemental_Files

## Data Availability

The 16S rRNA gene sequences generated in this study along with the metagenomic and metatranscriptomic datasets are deposited in the NCBI BioProject accession number PRJNA1320513. The 16S rRNA gene sequences used in this study were previously reported in our earlier works and can be accessed through the cited references [3, 50]. The genomes of newly enriched Cloacimonadota species have been deposited in SeqCode: the complete and closed genome of Ca. Digestoria delfossei is available in registry https://seqco.de/i:52909, and the high-quality draft genome of Ca. Cloacimonas fortuita is available in registry https://seqco.de/i:52907.
