# Supplementary material for: Phylum-wide propionate degradation and its potential connection to poly-gamma-glutamate biosynthesis in Candidatus Cloacimonadota phylum
Source: ISME J. 2026 Mar 18;20(1):wrag055. doi: 10.1093/ismejo/wrag055 (PMC13082233; doi:10.1093/ismejo/wrag055)
Supplement: wrag055_Supplemental_Files [file wrag055_supplemental_files.zip › Calusinska_et_al_Supplementary_Material_revised_wrag055.pdf]

Supplementary Material

Phylum-wide propionate degradation and its potential connection to poly-gamma-glutamate biosynthesis in *Candidatus* Cloacimonadota phylum

Magdalena Calusinska[1†], Malte Herold[1†], Dominika Klimek[1,2], Marie Bertucci[1], Sébastien Lemaigre[1], Sébastien Cambier[1], Simone Zorzan[1], Céline Leclercq[1], Jan Dolfing[3], Maria Westerholm[4], Bettina Müller[4], Leila Nasirzadeh[4,5], Anna Schnürer[4], Paul Wilmes[2,6], Philippe Delfosse[1,7] and Xavier Goux[1]

[1]Environmental and Industrial Biotechnology, Luxembourg Institute of Science and Technology, Hautcharage L-4940, Luxembourg

[2]The Faculty of Science, Technology and Medicine (FSTM), University of Luxembourg, Esch-sur-Alzette L-4365, Luxembourg

[3]Faculty of Energy and Environment, Northumbria University, Newcastle-upon-Tyne NE1 8QH, United Kingdom

[4]Department of Molecular Sciences, Swedish University of Agricultural Sciences, BioCentre, Uppsala SE-75007, Sweden

[5]Department of Biomedical and Clinical Sciences, The Division of Cell and Neurobiology, Linkoping University, Linkoping 581 83, Sweden

[6]Luxembourg Centre for Systems Biomedicine, University of Luxembourg, Esch-sur-Alzette, L-4365 Luxembourg

[7]Rectorate, Université du Luxembourg, Maison du Savoir, Esch-sur-Alzette L-4365, Luxembourg

† These authors contributed equally to this work

Supplementary Methods

1.1 **Anaerobic baffled reactors and experimental design**

Operation of reactors

Four three-compartment anaerobic laboratory-scale baffled reactors (ABRs) with a capacity of 100 L were inoculated with anoxic sludge from a full-scale methanogenic reactor at the wastewater treatment (WWTP) plant in Schifflange, Luxembourg. The ABRs operated at 37°C over a period of 174 days (Fig. 1), each reactor was compartmentalized by two baffles (each reactor compartment of 33L volume), with the first compartment (i.e., hydrolysis tank) receiving feed. The ABR reactors were manually fed with commercial dried sugar beet pulp (SBP) pellets following a semi-continuous feeding scheme, receiving feed on working days only, with no feeding on weekends (Fig. S1). Reactors were fed at an organic loading rate (OLR) increasing from 0.5 to 3 kg VS $m^{-3}d^{-1}$ for ABR0 (Fig. 1A, C, E) and an OLR of 0.5 to 6 kg VS $m^{-3}d^{-1}$ for ABR1-3 (Fig. S1). The pulp contained 0.012 grams of nitrogen (N) per gram of volatile solids ($g_N.g_{VS}^{-1}$), and its biochemical methane potential (BMP) was measured to be 0.38 normalized liters of CH_4 per gram of volatile solids (VS; $LN_{CH4}.g_{VS}^{-1}$), according to [1]. The hydraulic retention time (HRT) was adjusted to 56 days by adding tap water to the SBP pellet. A total of 7L of sludge was re-circulated from the last compartment to the first, (the hydrolysis tank), each time the reactors received feed (Fig. S1). Samples were regularly collected from each reactor compartment for analytical analyses. Metadata, including gas composition, pH, total ammonium nitrogen (TAN; g_N L_{sludge}^{-1}), total inorganic carbon (TIC; g_CaCO3 L_{sludge}^{-1}), total solids (TS; % of total solid/fresh sludge [w/w]), volatile solids (VS; % of volatile solids/% of total solids [w/w]), and volatile fatty acids (VFAs; $g.kg_{sludge}^{-1}$), were analyzed as previously described [1].

Biogas production was measured automatically every two hours, as previously described [1]. Methane content in the biogas was estimated using gas chromatography (CompactGC, Global Analyser Solutions, Interscience). Volatile fatty acids (VFAs), including formate, acetate, propionate, iso-butyrate, n-butyrate, and caproate, were monitored as described below. Briefly, approximately 250 µL of collected digestate was centrifuged at 15,000 x g for 5 minutes at 4°C. A total of 150 µL of the supernatant was collected and mixed with 600 µL of distilled water. Samples were filtered through PVDF 0.45 µm filters, and 50 µL of the collected filtrate was mixed with distilled water. Samples were stored at -20°C if not analyzed immediately. VFAs were measured by ion-exchange chromatography with conductivity detection and electrochemical suppression using an Ion Chromatograph ICS 5000 Dual-Channel from Thermo Fisher Scientific. VFAs were eluted with KOH on a Dionex IonPac AS18 column equipped with a guard column AG18.

Bioaugmentation trials

ABR2, also fed with SBP, was used in the bioaugmentation trials, whereas ABR3 was set as the negative bioaugmentation control (Fig. S1-S5). The aim of this experiment was to evaluate the potential application of the reactor microbiome enriched with Cloacimonadota OTU_1 as a remedy for treating acidified reactors with significant propionate concentrations. The fed compartment (hydrolysis tank) of the bioaugmented ABR2 was highly acidified, with a pH below 5.0, propionate concentrations above 5000 mg.kg^{-1} of sludge, and methane content in the produced biogas of less than 30% (Supplementary Dataset 1, Tables S1-S3). The bioaugmentation procedure began on day 132 and continued for 5 weeks. In total, five bioaugmentation events were applied, during which 3 L of sludge from the fed compartment of the ABR1 were transferred to the first compartment (hydrolysis tank) of the bioaugmented reactor (ABR2). ABR3 was not bioaugmented, and its first compartment did not enrich *Cloacimonadota* OTU_1 (Fig. S5).

1.2 Sequencing and correlation network analysis

Library preparation, sequencing, and data analysis

Macromolecules DNA/RNA were co-extracted using the Allprep DNA/RNA Mini kit (Qiagen, Hilden, Germany), following the manufacturer's instructions. The eluate was then divided into two parts: one part was treated with 1 µL of 10 µg/ml RNaseA (Sigma) for 30 minutes at room temperature, and the other part was treated with TURBO DNase (Invitrogen) according to the manufacturer's instructions, to obtain pure DNA and RNA fractions, respectively. The quality and quantity of extracted nucleic acids were assessed using the Bioanalyzer (Agilent) and Qubit (Invitrogen), respectively. DNA extracts were stored at -20°C until further library preparations. RNA extracts were stored at -80°C until further library preparations.

The bacterial and archaeal 16S rRNA gene amplicon libraries were sequenced using the MiSeq platform (Illumina), as previously described [2,3]. In summary, a modified version of universal bacterial primers S-D-Bact-0909-a-S-18 and S--Univ--1392-a-A-15, and archaeal primers S-D-Arch-0519-a-S-15 and S-D-Arch-1041-a-A-18 [4], together with the Nextera XT Index Kit V2 (Illumina), were used in a two-step PCR amplification. This amplified a fragment of approximately 484 bp, spanning the V6–V8 region of the bacterial 16S rRNA gene, and a fragment of around 526 bp, spanning the V4-V6 region of the archaeal 16S rRNA gene. Following sequencing, Usearch v.7.0.1090_win64 software was used for quality trimming (fastq-maxee 1, fastq_minlen 400 for bacteria and fastq_minlen 500 for archaea), chimera checking, removal of singletons, and assignment of sequences to operational

taxonomic units (OTUs) at the 97% similarity level, according to the pipeline described previously [5]. The taxonomic affiliation of the resulting OTUs was performed using the SILVA database v.138.2 [6] with mothur (v.1.38.0 [7]). The sequencing reads are available in the Sequence Read Archive (SRA) database under bioproject ID PRJNA1320513.

Associations between CLR-transformed (centered log-ratio) relative bacterial abundances derived from 16S rRNA gene amplicon sequencing and propionate concentrations at matching timepoints were explored separately for each reactor (1st compartment) using Spearman rank correlation (cor.test function in R, n = 10 per reactor). No correction for multiple testing was reported given the exploratory nature of this hypothesis-generating analysis and limited sample size. Due to the time-series nature of the data, temporal autocorrelation may violate independence assumptions. Therefore, results were interpreted with emphasis on effect size magnitude (Spearman's correlation coefficient, ρ) and biological plausibility rather than statistical significance alone.

Metagenomics and metatranscriptomics

Carbohydrate-active enzyme (CAZy) coding genes (CAZymes) were detected using dbCAN2 [8] and the CAZy database v6 [9].

Protein clusters and conservation scores

Protein cluster conservation was assessed using a Shannon entropy-based approach. For each cluster containing at least 100 member sequences, a multiple sequence alignment was generated using MAFFT v7.526 with automatic strategy selection (--auto). Conservation scores were subsequently computed using a custom Python 3.13 script. For each alignment column, the Shannon entropy was calculated as H = -sum(f_a * log2(f_a)), where f_a is the observed frequency of amino acid a in that column. Columns in which the fraction of gap characters exceeded 0.3 were excluded from the analysis. The per-column conservation score was defined as the complement of Shannon entropy relative to the theoretical maximum for 20 amino acids (log2(20) ≈ 4.32 bits), such that higher scores reflect greater conservation. A cluster-level conservation score was obtained by averaging the per-column scores across all retained alignment positions.

Correlation network calculation

Correlation network analysis (CNA) was performed using two datasets (combined) containing sequences corresponding to the Cloacimonadota OTU_1. The first dataset comprised sequences analyzed in this study. The second dataset corresponded to the amplicon sequence variant (ASV)-

level data previously published [10]. For both datasets calculation was done at the ASV level (not shown), and bacterial and archaeal sequences were incorporated into the analysis to capture potential interactions across the microbial community. The method for calculating pairwise correlations and data filtration was adapted from [11]. To enhance the sensitivity of resulting networks, infrequent ASVs were filtered out, retaining only those representing at least 0.1% of the total community abundance, as previously recommended [12]. Subsequently, pairwise Spearman's rank correlations were computed, and the resulting correlation *p*-values were corrected for multiple comparisons using the Benjamini-Hochberg correction. Data filtering retained only highly significant correlations, defined as those with a P value ≤ 0.001 and an R coefficient ≥ 0.5. Initially, general correlation networks were constructed. To identify potential syntrophic partners of Cloacimonadota OTU_1, direct neighborhood correlation networks were then constructed using only OTUs/ASVs highly positively or negatively correlated with our Cloacimonadota species of interest. Topological features such as Degree Centrality, Betweenness Centrality, Closeness Centrality, and Eigenvector Centrality were calculated for each microbial correlation network to identify potential key actors (i.e., keystone ASVs; Supplementary Dataset 1, Table S4). All calculations were performed using R version 3.6.1, RStudio version 1.1.383, Cytoscape version 3.7.2, along with the R Bioconductor package RCy3 version 2.4.4 and the R package igraph version 1.2.4.1.

1.3 Metaproteomics

Two samples (Fig. 1), including technical duplicates, were centrifuged at 4°C at 10 000 g for 20 min in an Allegra 64R centrifuge (Beckman Coulter, USA). The pellet was suspended in 600 µl of SDS buffer (30% sucrose, 2% sodium dodecyl sulphate (SDS), 0.1 M Tris-hydrochloride (HCl), 5% β-mercapto-ethanol, pH = 8), vortexed and incubated at 65°C for 1 h 30. After incubation, the tube was filled with phenol buffer (Invitrogen, Thermofisher scientific) at room temperature, vortexed for 30 s and centrifuged at 10,000 *g* for 3 min. Three phases (lower phase = phenol + cellular debris, upper phase = aqueous SDS phase with solubilized proteins and fat) were formed in the tube and the upper phase was extracted into a new tube. The extract was diluted in cold 0.1 M ammonium acetate in methanol and kept at −20°C for the night, after which it was centrifuged at 10,000 *g* for 5 min. The pellet was washed twice with cold acetone and dried. Finally, the dried pellet was solubilized in 50 µl lysis buffer (7 M urea, 2 M thiourea, 0.5% (*w*/*v*) CHAPS). Protein concentration was determined following the RC DC™ (reducing agent, detergent compatible) protein assay (Bio-Rad) with bovine serum albumin (BSA) for the standard curve. Samples were kept at −20°C until further analysis.

20 µg of total proteins were loaded and separated on a Criterion ™ XT precast 1D-gel (4‑12% bis-tris, 1.0 mm × 12 wells, Bio-Rad, USA) following manufacturer's instructions. After a short migration, gels were stained, cut into small pieces for each sample to perform in-gel digestion. Each sample was reduced, alkylated and destained. Then, proteins were digested using trypsin enzyme (sequencing mass grade, Promega, USA). The extracted peptides were analysed with a NanoLC 425 Eksigent coupled to a TripleTOF® 6600 MS (Sciex, Belgium). Peptides were loaded onto the trap column (C18 acclaim™ PepMap™, 5 µm, 5 mm × 300 µm, Thermo Scientific, Germany) and desalted for 5 min at a flow rate of 2 µl/min using loading buffer (2% v/v acetonitrile, 0.05% (v/v) trifluoroacetic acid in water LC-MS grade). After this, peptides were separated onto a C18 reverse phase column at a flow rate of 300 nl/min (C18 acclaim™ PepMap™ 100, 3 µm, 100 A, 75 µm × 15 cm, Thermo Scientific, Bremen, Germany) using a binary gradient (solvent A: H_2O LC-MS, 0.1% (v/v) formic acid; solvent B: acetonitrile, 0.1% (v/v) formic acid). Peptides were eluted from 3% B to 30% over 60 min, increased to 40% B during 10 min then increased to 80% B until 10 min, and then re-equilibrated prior to the next injection for 20 min at 3% B. MS scan was followed by 30 MS/MS scans from 300 to 1250 m/z with 250 ms of accumulation time and from mass range 100–1500 m/z with 50 ms of accumulation time respectively using the automatically adjusted system of rolling collision energy voltage.
The acquired MS and MS/MS data were imported into Progenesis QI for Proteomics software (version 4.2, Nonlinear Dynamics, Waters). Then the protein and peptide identification were imported to Progenesis QIP, searching against *Ca.* Digestoria delfossei genome and the reconstructed Methanothrix sp genome draft (bin.28; Supplementary Dataset 2, Table S5) via Mascot Daemon (version2.6.0. Matrix Science, UK) and matched to peptide spectra. The following Mascot research parameters were used: peptide tolerance of 20 ppm, fragment mass tolerance of 0.5 Da, a maximum of two missed cleavages, carbamido-methylation of cysteine as fixed modification and oxidation of methionine, N-terminal protein acetylation and tryptophan to kynurenine as variable modifications. Only the proteins identified with a significance Mascot-calculated confidence of 95% and at least two sequences and one unique sequence per protein were accepted. Results are provided in Supplementary Dataset 3, Table S14.

1.4 Enrichment and isolation trials

The initial enrichment of bacteria from anaerobic digestion (AD) systems was part of a larger study investigating microbial communities in these environments (details not described here). Two inocula were used, including thickened material from AD systems fed with either activated sludge or agricultural biowaste. Before media inoculation, the sludge inocula were pre-treated with broad-

spectrum antibiotics, including vancomycin (see below) and diluted 10- or 100-fold. From these enrichments, 21 promising cultures that retained the presence of Cloacimonadota were selected for further tailored cultivation. To promote the growth of Cloacimonadota, various media and cultivation conditions were tested, at neutral pH and incubation temperatures of room temperature or 37°C (Supplementary Dataset 5). Given that Cloacimonadota are hypothesized to produce extracellular polymeric substances composed of poly-gamma-glutamate (PGA), we speculated that this feature could enhance resilience to environmental stressors by acting as a protective layer, including against antibiotics. As a result, despite the putative resistance of Cloacimonadota to vancomycin (data not shown), a combination of broad-spectrum antibiotics (ampicillin, ciprofloxacin, streptomycin, and vancomycin) efficiently suppressed the growth of competing bacteria (Supplementary Dataset 5, Table S16). At the end of the incubation period, DNA was extracted from the samples for microbial community analysis using 16S rRNA gene amplicon sequencing and metagenomics.

1.5 Fluorescence *in situ* hybridization and microscopy

Flock formation was observed within the culture media, which could be disrupted by vigorous shaking. To enable microscopic visualization of physical associations between Cloacimonadota and its putative syntrophic partner *Methanothrix*, culture samples were handled gently to minimize disturbance and avoid centrifugation steps. Floating flocks were harvested using a sterile syringe and needle, fixed in 4% paraformaldehyde (w/v) at a 1:3 ratio, and incubated for 3 hours at 4°C. After fixation, samples were washed twice in PBS buffer and stored in a PBS (1:1) solution at -20°C until further processing. Between 5 to 10 µl of sample was applied onto a 10-well FISH slide and air-dried overnight. Subsequently, slides were dehydrated using a series of ethanol solutions with concentrations of 50%, 80%, and 100% for 3 minutes each, followed by air-drying. The hybridization buffer with a 40% formamide concentration was pre-warmed and applied to the slide. Probes were then added at a final concentration of 4.5 pmol.μl^{-1}. Slides were placed in a 50-mL Falcon tube containing moistened paper and incubated horizontally in a hybridization oven for 2 hours at 46°C. After hybridization, slides were washed with the wash buffer at 48°C for 25 minutes. For microscopy, a DAPI-containing antifade mounting solution (Fluoroshield with DAPI, Merck) was applied to the slide and covered with a coverslip. Visualization was performed using a Zeiss LSM 880 system coupled with an inverted Zeiss Axiovert 200 M microscope (Zeiss, Jena, Germany) for image acquisition. Image processing and visualization were conducted using Zeiss ZEN 2011 software.

Supplementary Results and discussion

2.1 Bioaugmentation assays

The bioaugmentation procedure began on day 132 (Fig. S5). Following this, *Ca.* Digestoria delfossei (OTU_1) successfully established itself within the microbial community of the bioaugmented reactor ABR2, whereas no change was observed in the control not-bioaugmented ABR3. Propionate consumption started within just a few days of starting the bioaugmentation in ABR2, leading to a gradual pH increase. By day 146 (14 days after the procedure began), the reactor pH had returned to neutral, and the biogas produced contained over 50% CH_4 (Fig. S2). This restoration of anaerobic digestion demonstrates the effectiveness of bioaugmentation in mitigating reactor acidosis.

2.2 Correlation network analysis

The dataset generated in this study merged with the previous dataset [1] included 282 samples for a total of 8955 ASVs. Around 98.2% of these ASVs were from bacterial origin, whereas archaea represented only 1.8% of the studied community. Only 154 ASVs were kept after the first filtering (i.e., representing more than 0.1% of the total microbial community) for the next step (representing 1.7% of the initial number of ASVs). Following the Spearman's pairwise rank correlation calculation, a total of 2369 interactions having an adjusted p-value ≤ 0.001 and a R coefficient ≥ 0.5 (1709 positives (72.1%) and 660 negatives (27.9%)) were established between the previously selected 154 ASVs (100 were of bacterial origin (64.9%) and 54 of archaeal origin (35.1%)).

The Cloacimonadota OTU_1 (B_ASV1) was included in a direct neighbor correlation network including 35 ASVs (26 from bacterial origin (74.3%) and 9 from archaeal origin (25.7%)) and 309 interactions (238 positives (77.0%) and 71 negatives (23.0%)). Topological features, such as Degree, Betweenness, Closeness and Eigenvector centrality were used to identify potential specific keystone ASVs (Supplementary Dataset 1, Table S4). The first ASV highlighted as potential key partners of the *Ca.* Digestoria delfossei OTU_1 (represented as B_ASV1) was from archaeal origin (A_ASV4) and it was taxonomically affiliated to *Methanothrix sp*. A second archaeon (A_ASV11) was taxonomically affiliated with the *Methanosarcina* genus.

2.3 The presence of other, potentially novel syntrophic propionate oxidizing bacteria in the ABR1

To assess the presence of other putative syntrophic propionate oxidizing bacteria (SPOB), we further analyzed reconstructed genomes within ABR1. We identified two metagenome assemble genomes

(MAGs), including bin30 and bin45, both classified within the *Bacteroidota*, which encoded a potentially complete methyl malonyl-CoA (mmc) pathway, suggesting they may represent either previously uncharacterized SPOB, alternatively propionate producers (Supplementary Dataset 3, Table S14; Fig. S8). However, most of the associated genes were scattered throughout the genome and not organized into a typical *mmc* cluster, as previously described for the known SPOB [13]. Additionally, a few other MAGs encoded components of the Cloacimonadota-specific *mmc* pathway (Fig. S8). Given the presence of these microbes, we sought to determine whether the novel *Ca.* Digestoria delfossei would still play a role in propionate consumption and whether it might employ a yet-undescribed metabolic pathway (see below). To address this, we briefly analyzed the metatranscriptomic abundance of known mmc genes across the different MAGs, including the novel *Ca.* Digestoria delfossei. We observed that whereas the *mmc* genes were expressed by various MAGs during different experimental phases, their overall expression levels were very low, including *Ca.* Digestoria delfossei (Supplementary Dataset 3, Table S13). This may be attributed to the disproportionately high expression of a few archaeal genes involved in methane production, which accounted for a significant proportion of the mapped reads in our metatranscriptomic dataset (Fig. S7). Consequently, much deeper sequencing would have been required to adequately capture the expression patterns of genes with lower metatranscriptomic abundance. As a result, the metatranscriptomic analysis provided only limited insights, primarily confirming that all presumed SPOB expressed their *mmc* genes at some point during the experiment. A more detailed investigation of these other SPOB was beyond the scope of this study.

2.4 Database of Cloacimonadota genomes and AD type-specific separation of Cloacimonadota clades

The majority of Cloacimonadota MAGs are sourced from aquatic environments (sea and lake samples and sediments) and terrestrial methanogenic habitats (e.g., anaerobic digestion reactors, landfills, activated sludge, sedimentary rocks), and fewer with host-associated origins including the termite gut (Supplementary Dataset 2, Table S7). Clades I (represented by 153 MAGs), III (105 MAGs) and IV (121 MAGs) contained species representative of AD and WWTP environments (78%, 74%, 26% respectively), at the same time regrouping the highest number of reconstructed MAGs. Clade II contained 179 MAGs from aquatic habitats, including MAGs of marine origin, such as deep-sea sediments and hydrothermal vents and freshwater. It regrouped the highest diversity of candidate species (103 genome clusters – GCs) that were mainly represented by single MAGs (77 GCs). Two smaller clusters within clade II contained MAGs originating from the termite gut studies (GC-149, GC-

8) and ground water and sedimentary rocks (GC-11, 55, 68, 90), both regrouping the lowest number of MAGs, entirely representing separate candidate species (i.e., genome clusters). Genome size distribution (between 1.2 to 4 Mb), number of encoded proteins (1.2 to 3.4 k) and the GC content (32 to 56%) highlighted a significant genetic variation within Cloacimonadota clades (Fig. S9). However, no clear clade-specific trends were revealed, except for the GC content distribution, for which differences between clades were evident.
Both clades I and III encompassed the majority of representatives originating from diverse anaerobic digestion systems. However, further 16S rRNA gene comparison (whenever the 16S rRNA gene was present in a MAG) with the previous study analyzing the abundance and stability of microbial communities in full scale energy units [3], showed that species from clade I were commonly abundant in diverse types of AD units (i.e., farm reactors receiving manure and mixed biowaste fed units). Whereas Cloacimonadota from clade III dominated mainly ADs operating at WWTPs. This apparent separation is in accordance with the source of metagenomes that were at the origin of the different MAGs (Supplementary Dataset 2, Table S7).

2.5 Protein clusters and hypothetical proteins in Cloacimonadota genomes

Over half of the proteins could not be functionally annotated through whole-sequence searches against existing databases (43.6% of 967,210 protein sequences annotated in GhostKoala), limiting our ability to perform an unbiased comparison of Cloacimonadota metabolic capacities. To address this, we grouped homologous proteins by clustering all 967,210 proteins predicted from the analyzed Cloacimonadota genomes into protein clusters (PCs). This clustering yielded 68,098 PCs, including 39,402 singletons (representing 4.0% of all proteins), which are predicated protein sequences with no homologs in distinct Cloacimonadota genomes. A total of 6,619 PCs, representing 44.6% (431,099) of proteins (along with their protein conservation scores), were assigned KOs functionalities through the cluster representative sequences (Supplementary Dataset 2, Table S9), leaving a substantial portion of proteins classified as hypothetical.

To further investigate functional diversity, PCs were assigned to KEGG Orthologies (KOs) to evaluate subfamily variation among functionally assigned PCs. Overall, no correlation was observed between the diversity (number of PCs) associated with a KO category and the total number of genes assigned to that category. This suggests that whereas certain functions are evolutionarily conserved within the Cloacimonadota phylum, others exhibit considerable variation (data not shown). Among the top 100 most abundant PC-KOs, 20 were represented by single PCs, nearly half by two PCs, and over 80% by just five PCs (Supplementary Dataset 2, Table S9). We hypothesize that functions encoded by

proteins assigned to the most abundant KOs but represented by single PCs are likely of fundamental importance to the phylum's activity. These functions, being widespread yet highly conserved (i.e., relatively unchanged at the sequence level), likely play a critical role in Cloacimonadota metabolism. For example, three of the most abundant KOs represented by single PCs include phosphate butyryltransferase (likely propionyltransferase; see main text for details), methylmalonyl-CoA decarboxylase subunit alpha, and the type IV pilus assembly protein PilB. The first two are components of a propionate oxidation pathway, whereas the presence of PilB suggests a potential role in direct electron transfer (DIET), a characteristic also observed in some other SPOB [13]. Additional genes involved in the methylmalonyl-CoA pathway were among the most abundant and conserved PCs, further supporting the hypothesis that propionate oxidation is a phylum-wide capability of Cloacimonadota.

In contrast, 21 KOs were represented by more than 20 PCs, and among others they included different components of the type 1 restriction enzyme system (e.g., K01154, K03427, K01153), putative transposases and integrases (K07497, K04763), enzymes involved in nucleic acid (e.g., K03657, K00558, K04763, K03088, K03686) and amino acid metabolism (e.g., K12132, K04771, K01953) as well as some uncharacterized proteins (K07126, K06871, K07133).

2.6 Carbohydrate active enzymes in Cloacimonadota genomes

Previously, Cloacimonadota from engineered environments were found to encode a larger repertoire of carbohydrate active enzymes (CAZymes) compared to those in natural systems [14]. However, these primarily include multiple glycoside transferase coding genes (Supplementary Dataset 2, Table S10). In contrast, the diversity and number of hydrolysis CAZyme gene copies are much lower in Cloacimonadota compared to, for example, *Bacteroidota* or *Bacillota* (former Firmicutes) [15], suggesting their contribution to carbohydrate degradation in AD systems is rather limited. In clade I AD Cloacimonadota, multiple CAZymes are organized into two gene complexes which respectively target starch (α-glucans) and mannose (including acetyl-D-hexosamine) residue-containing carbohydrates. In the case of clade III only the former CAZyme complex was present. For comparison, no specific CAZyme gene complexes were identified in most of the aquatic Cloacimonetes. However, the reconstructed genomes were more fragmented, which might have impacted the search results.

2.7 Unique metabolic contributions of Cloacimonadota within anaerobic digestion communities

Reasoning that ubiquity indicates essential gene functions, while abundance combined with restricted phylogenetic distribution may reflect lineage-specific adaptations that could contribute to ecological specialization, we mainly focused on functions (i.e. KOs) predominantly present in Cloacimonadota (Fig. 4B; Supplementary Dataset 4, Table S15). As a result, we found only eleven KOs that were ubiquitous (>=80%) in all AD bacterial genomes but nearly absent (<= 5%) from Cloacimonadota. These KOs were mainly involved in cell biogenesis and phosphate starvation inducible stress (K06217). The latter enables cells to use limited phosphate resources more effectively [16]. We identified 56 KOs (35 with enzymatic function) nearly exclusively found in Cloacimonadota (found in at least 75% of AD Cloacimonadota vs. at maximum 25% of other AD bacteria), including those related to sodium (K09697) and phosphonates (K02041, K02042, K02044) transport, various metallo- (K06972, K05823) and serine-peptidases (K03503, K08676), uncharacterized proteins (K09129, K09141, K09798, K09729, K09005) and multiple KOs involved in lipopolysaccharide biosynthesis. The specialization of Cloacimonadota towards the plausible utilization of phosphonates, organophosphorous compounds with C-P bonds, as an alternative phosphorous source, might indicate that this phylum has carved out a specific niche within the AD community by utilizing a resource that is only marginally accessible to other organisms [17]. Indeed, sugar beet pulp has high fiber and protein content but low phosphorus content, which could stimulate the overdominance of Cloacimonadota in our ABR reactor fed with high OLR of this substrate (Fig. 1). However, this observation remains to be further studied.

2.8 Enrichment and cultivation of Claocimonadota from full-scale anaerobic digestion reactors

Enrichment and cultivation trials

Based on 16S rRNA gene analysis, one enrichment (RT_BT_as_ph7; Supplementary Dataset 5, Table S16) achieved a high dominance of a single OTU belonging to Cloacimonadota, comprising 56% of its relative abundance (Supplementary Dataset 5, Table S17). This enrichment was cultivated in PYGV medium (DSMZ 621 basal medium) supplemented with ampicillin (100 mg/L), streptomycin (100 mg/L), and D-glucose (1% w/v). Subsequent re-culture on PYGV medium supplemented with propionate (10% w/v) further stimulated the growth of the Cloacimonadota OTU (Supplementary Dataset 5, Table S18). This sample was subsequently used for metagenomic reconstruction (Supplementary Dataset 5, Table S19). Sequencing and analysis revealed that the enriched Cloacimonadota OTU represented a distinct species, which we later named *Ca.* Cloacimonas fortuita.

Additionally, the most abundant archaeal species in the enrichment was *Methanothrix* sp. (Fig. 5), supporting its putative role as a syntrophic partner of Cloacimonadota in AD reactors (Supplementary Dataset 1, Table S4).

Despite repeated transfers of the enriched culture to fresh medium, enriched Cloacimonadota cells were consistently lost (significant decrease in abundance), suggesting the absence of an essential factor present in the original sludge. To optimize conditions for isolation, 14 different media formulations and growth conditions were further tested, informed by genomic analyses of Cloacimonadota functional traits in AD environments. Additives such as acetate, propionate, pyruvate, potassium chromate, magnetite, and L-glutamate were included as potential growth promoters (Supplementary Dataset 5, Table S18). However, these efforts did not yield successful isolation of the target species (details not discussed here). The failure to isolate *Ca.* Cloacimonas fortuita may be attributed to stochastic fluctuations or the growth dependency on its archaeal symbiont *Methanothrix*, which appears crucial for its survival, under our experimental conditions. Other enrichments cultivated in the tested media and conditions also contained OTUs belonging to Cloacimonadota (Supplementary Dataset 5, Table S17), but none reached the high abundance observed in the dominant OTU enrichment. These results highlight the intricate ecological and physiological requirements of Cloacimonadota and underscore the challenges associated with isolating and maintaining members of this phylum in laboratory conditions.

Designation of two new *Cloacimonadota* species

***Candidatus* Digestoria delfossei**

The genome of the enriched bacterium in the ABR1 reactor was analyzed and compared to its closely related species, previously described *Ca.* Syntrophosphaera thermopropionivorans (GCA_004353895.1). ANI and AAI comparative analyses revealed values of 66.5% ANI and 66.4% AAI, respectively. These results surpassed the species-level threshold of 95% ANI placing the bacterium beyond the genus-level range of AAI (below 70%). Additionally, both genomes have largely different GC content, which is usually conserved within the same genus, reinforcing the likelihood that genome of the enriched bacterium represents a new genus. However, due to the lack of precise AAI cut-offs for *Ca.* Cloacimonadota, further studies, including full-length 16S rRNA gene comparisons, should complement these findings. Together, these findings indicated that the enriched Cloacimonadota species likely represent a new species within a genus distinct from *Ca.* S. thermopropionivorans. Based on genomic analysis, we propose it serves as the nomenclatural type

for the novel species and genus *Candidatus* Digestoria delfossei gen. nov. sp. nov. This species has been registered in SeqCode (https://seqco.de/i:52909). All associated higher taxa are left unnamed currently, pending further resolution of the taxonomy of the phylum Cloacimonadota.

Candidatus Digestoria gen. nov. (Di.ges.to´ri.a. L. fem. n. digestio, digestion; N.L. fem. n. Digestoria, a genus name referring to its role in anaerobic digestion).

Candidatus Digestoria delfossei sp. nov. (del.fos'se.i. N.L. gen. n. delfossei, named in honor of our former group leader, Dr. Philippe Delfosse).

***Candidatus* Cloacimonas fortuita**

The genome of the enriched bacterium in the culture RT_BT_as_ph7 was analyzed and compared to the known *Ca.* Cloacimonas acidaminovorans (GCA_000146065.1). Genomes of the enriched species and the closely related species *Ca.* C. acidaminovorans have an ANI of 78.3%, which is below the species-level cut-off (95%) but above the genus-level threshold (>70%). Their AAI value of 79% further supports that they belong to the same genus, *Ca.* Cloacimonas. Therefore, we propose the newly recovered genome as the nomenclatural type for the novel species within the Cloacimonas genus, and name it as *Candidatus* Cloacimonas fortuita sp. nov. This species has been registered in SeqCode (https://seqco.de/i:52907).

Candidatus Cloacimonas fortuita (for.tu.i'ta, L. fem. adj. fortuita, accidental, fortuitous, by chance; referring to the accidental enrichment of the strain).

Reference list

1. Lemaigre, S. *et al.* Potential of acetic acid to restore methane production in anaerobic reactors critically intoxicated by ammonia as evidenced by metabolic and microbial monitoring. *Biotechnol. Biofuels Bioprod.* **16**, 1–20 (2023).

2. Goux, X. *et al.* Microbial community dynamics in replicate anaerobic digesters exposed sequentially to increasing organic loading rate, acidosis, and process recovery. *Biotechnol. Biofuels* **8**, 1–18 (2015).

3. Calusinska, M. *et al.* A year of monitoring 20 mesophilic full-scale bioreactors reveals the existence of stable but different core microbiomes in bio-waste and wastewater anaerobic digestion systems. *Biotechnol. Biofuels* **11**, 1–19 (2018).

4. Klindworth, A. *et al.* Evaluation of general 16S ribosomal RNA gene PCR primers for classical and next-generation sequencing-based diversity studies. *Nucleic Acids Res.* **41**, 1–11 (2013).

5. Edgar, R. C. Search and clustering orders of magnitude faster than BLAST. *Bioinformatics* **26**, 2460–2461 (2010).

6. Yilmaz, P. *et al.* The SILVA and 'all-species Living Tree Project (LTP)' taxonomic frameworks. *Nucleic Acids Res.* **42**, 643–648 (2014).

7. Schloss, P. D. *et al.* Introducing mothur: Open-source, platform-independent, community-supported software for describing and comparing microbial communities. *Appl. Environ. Microbiol.* **75**, 7537–7541 (2009).

8. Zhang, H. *et al.* DbCAN2: A meta server for automated carbohydrate-active enzyme annotation. *Nucleic Acids Res.* **46**, W95–W101 (2018).

9. Drula, E. *et al.* The carbohydrate-active enzyme database: Functions and literature. *Nucleic Acids Res.* **50**, D571–D577 (2022).

10. Lemaigre, S. *et al.* Potential of multivariate statistical process monitoring based on the biogas composition to detect free ammonia intoxication in anaerobic reactors. *Biochem. Eng. J.* **140**, 17–28 (2018).

11. De Vrieze, J., Pinto, A. J., Sloan, W. T. & Ijaz, U. Z. The active microbial community more accurately reflects the anaerobic digestion process: 16S rRNA (gene) sequencing as a predictive tool. *Microbiome* **6**, 63 (2018).

12. Berry, D. & Widder, S. Deciphering microbial interactions and detecting keystone species with

co-occurrence networks. *Front. Microbiol.* **5**, 1–14 (2014).

13. Westerholm, M., Calusinska, M. & Dolfing, J. Syntrophic propionate-oxidizing bacteria in methanogenic systems. *FEMS Microbiol. Rev.* **46**, 1–26 (2022).

14. Johnson, L. A. & Hug, L. A. Cloacimonadota metabolisms include adaptations in engineered environments that are reflected in the evolutionary history of the phylum. *Environ. Microbiol. Rep.* **00**, 1–10 (2022).

15. Campanaro, S. *et al.* New insights from the biogas microbiome by comprehensive genome-resolved metagenomics of nearly 1600 species originating from multiple anaerobic digesters. *Biotechnol. Biofuels* **13**, 1–18 (2020).

16. Antelmann, H., Scharf, C. & Hecker, M. Phosphate starvation-inducible proteins of Bacillus subtilis: Proteomics and transcriptional analysis. *J. Bacteriol.* **182**, 4478–4490 (2000).

17. Sosa, O. A. *et al.* Isolation and characterization of bacteria that degrade phosphonates in marine dissolved organic matter. *Front. Microbiol.* **8**, 1–16 (2017).

Supplementary Figures

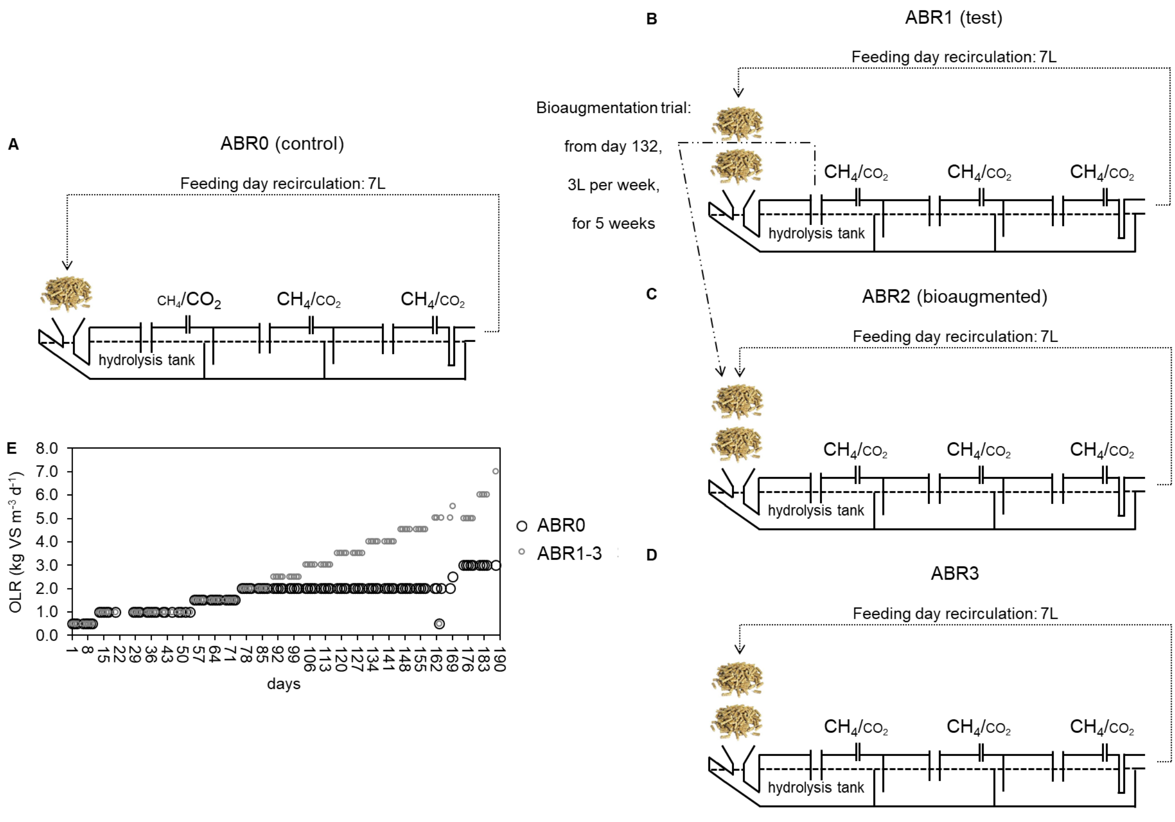

Figure S1. A detailed feeding and sludge recirculation plan implemented for the anaerobic baffled reactors (ABRs), encompassing the ABR0 (A), ABR1 (B), the bioaugmented ABR2 (C) and ABR3 (not-bioaugmentation control; D).

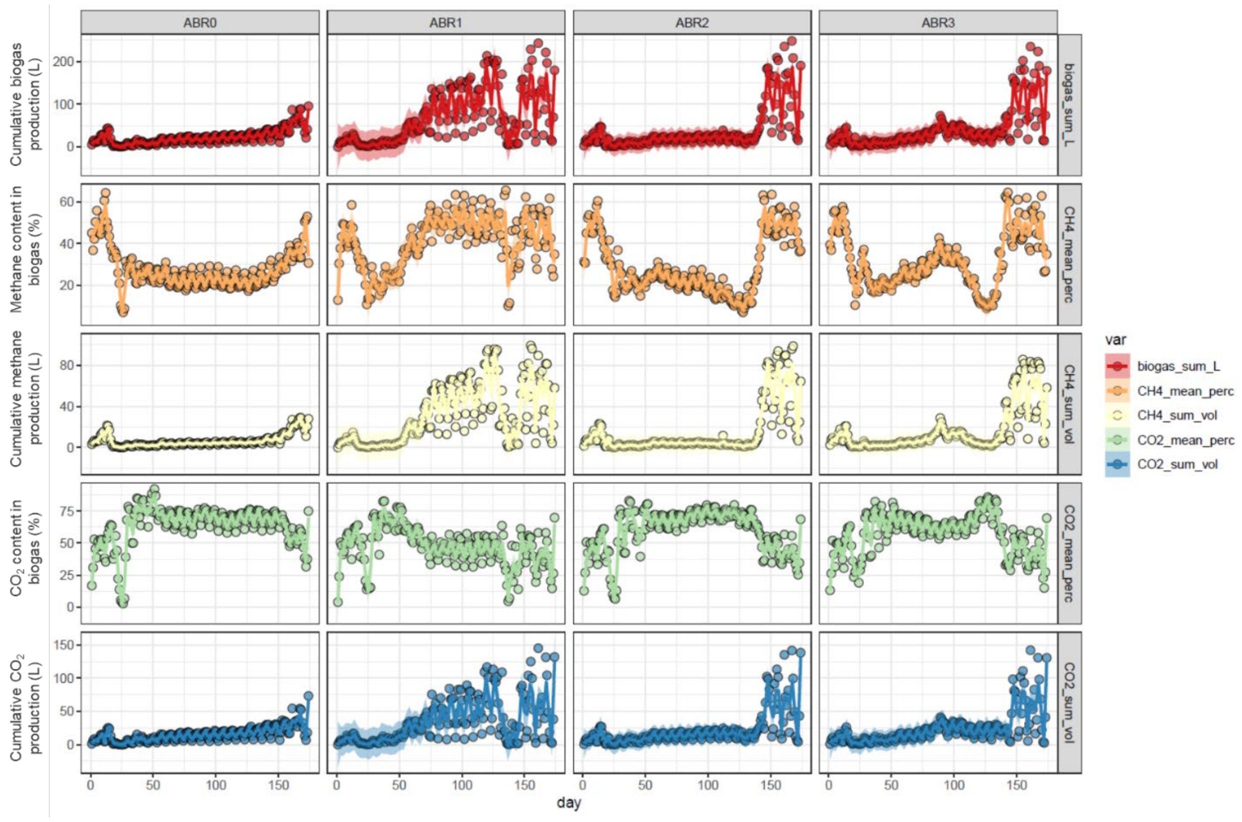

Figure S2. Biogas, methane and CO_2 production profiles in the hydrolysis tank (first compartment) for the different ABR reactors.

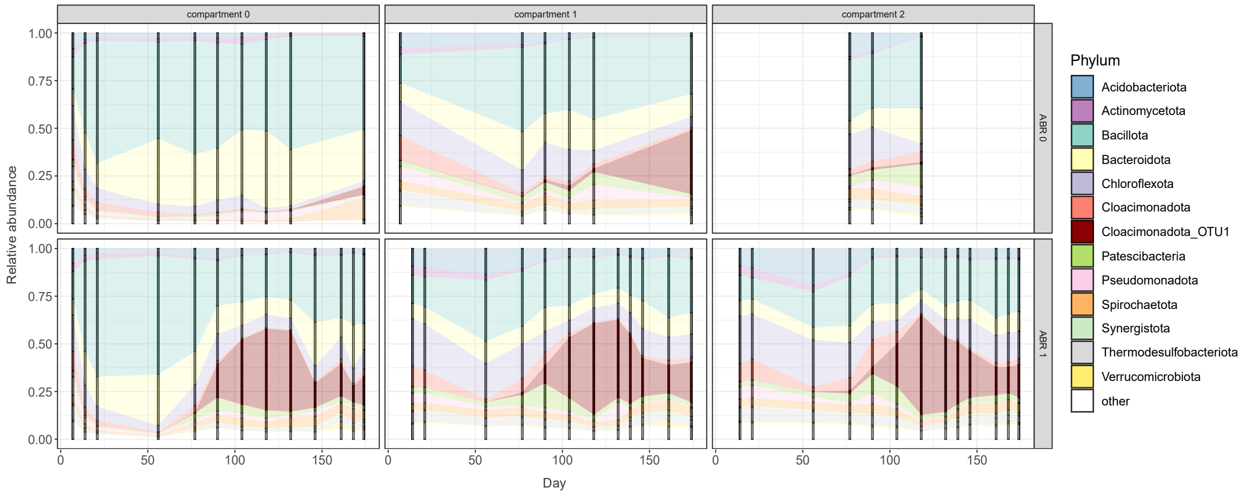

Figure S3. Phylum-level bacterial community composition in the ABR0 and the ABR1 across the different reactor compartments, as determined by 16S rRNA gene amplicon sequencing (Supplementary Material, Table S2). Missing data points are due to failed sequencing samples.

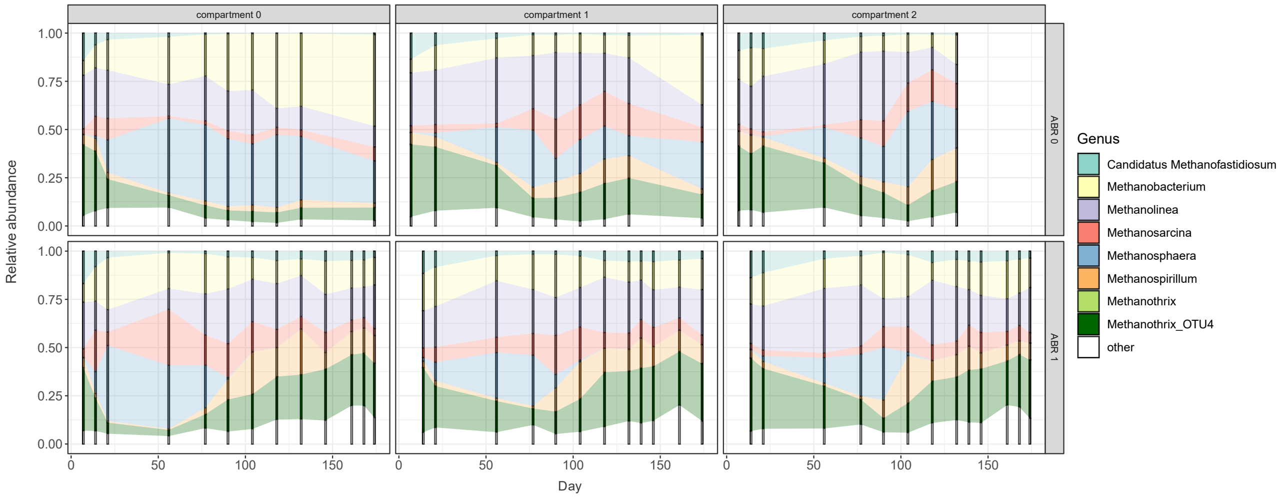

Figure S4. Genus-level archaeal community composition in the ABR0 and the ABR1 across the different reactor compartments, as determined by 16S rRNA gene amplicon sequencing (Supplementary Material, Table S3). Missing data points are due to failed sequencing samples.

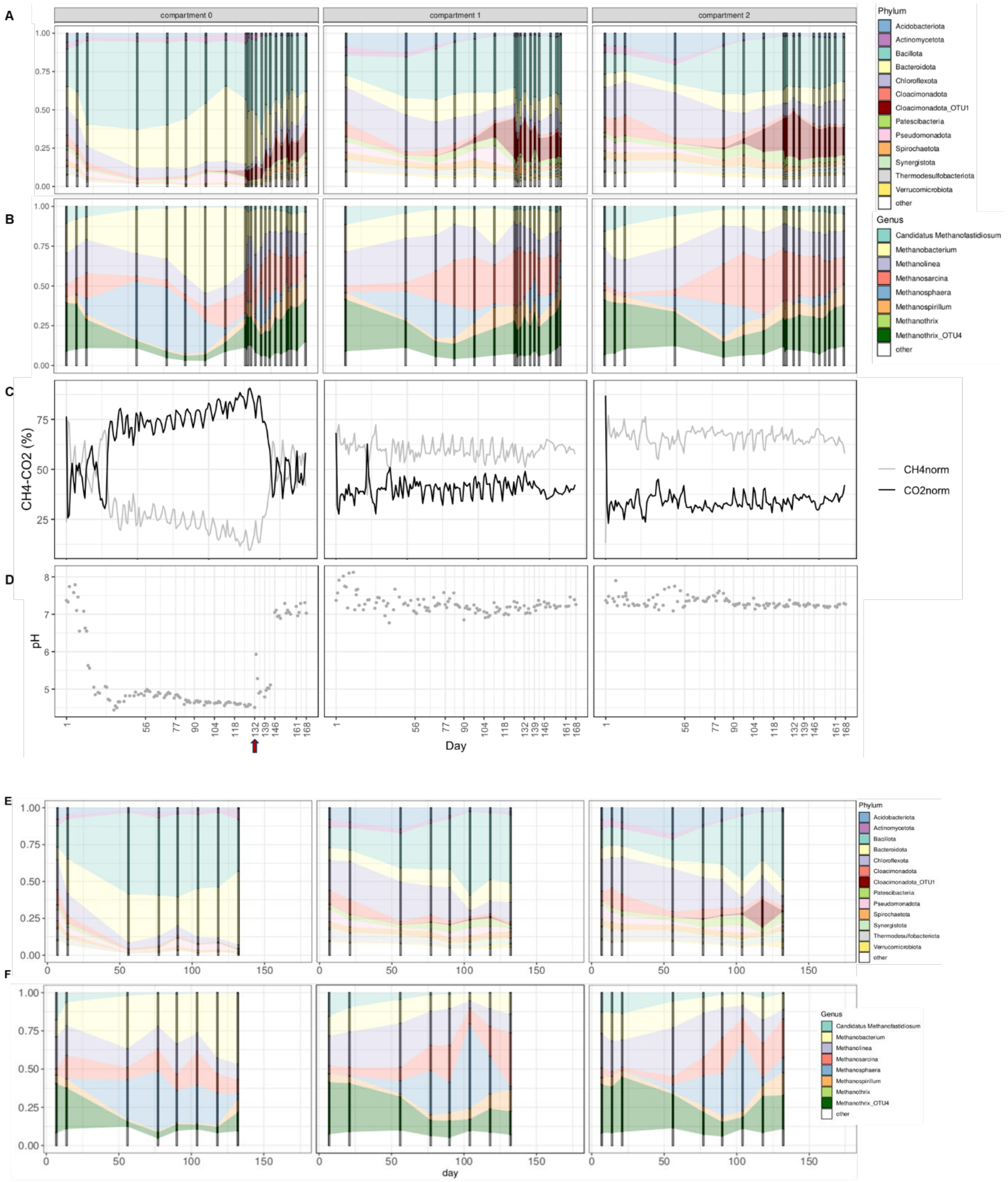

Figure S5. Bioaugmentation trials of the acidified anaerobic baffled reactor (ABR2; A-D) using Cloacimonadota OTU_1-enriched sludge from the ABR1. ABR3 was used a control, non-bioaugmented reactor (E-F). Phylum-level bacterial (A) and genus-level archaeal (B) community composition in ABR2 across the different compartments; percentages of CH_4 and CO_2 in the biogas produced by ABR2 (C); pH values and propionate concentrations in ABR2 (D). The black arrow marks day 132, indicating the start of bioaugmentation with sludge enriched in *Ca.* Cloacimonadota OTU_1.

Phylum-level bacterial (E) and genus-level archaeal (F) community composition in ABR3 (non-bioaugmented control) across the different compartments

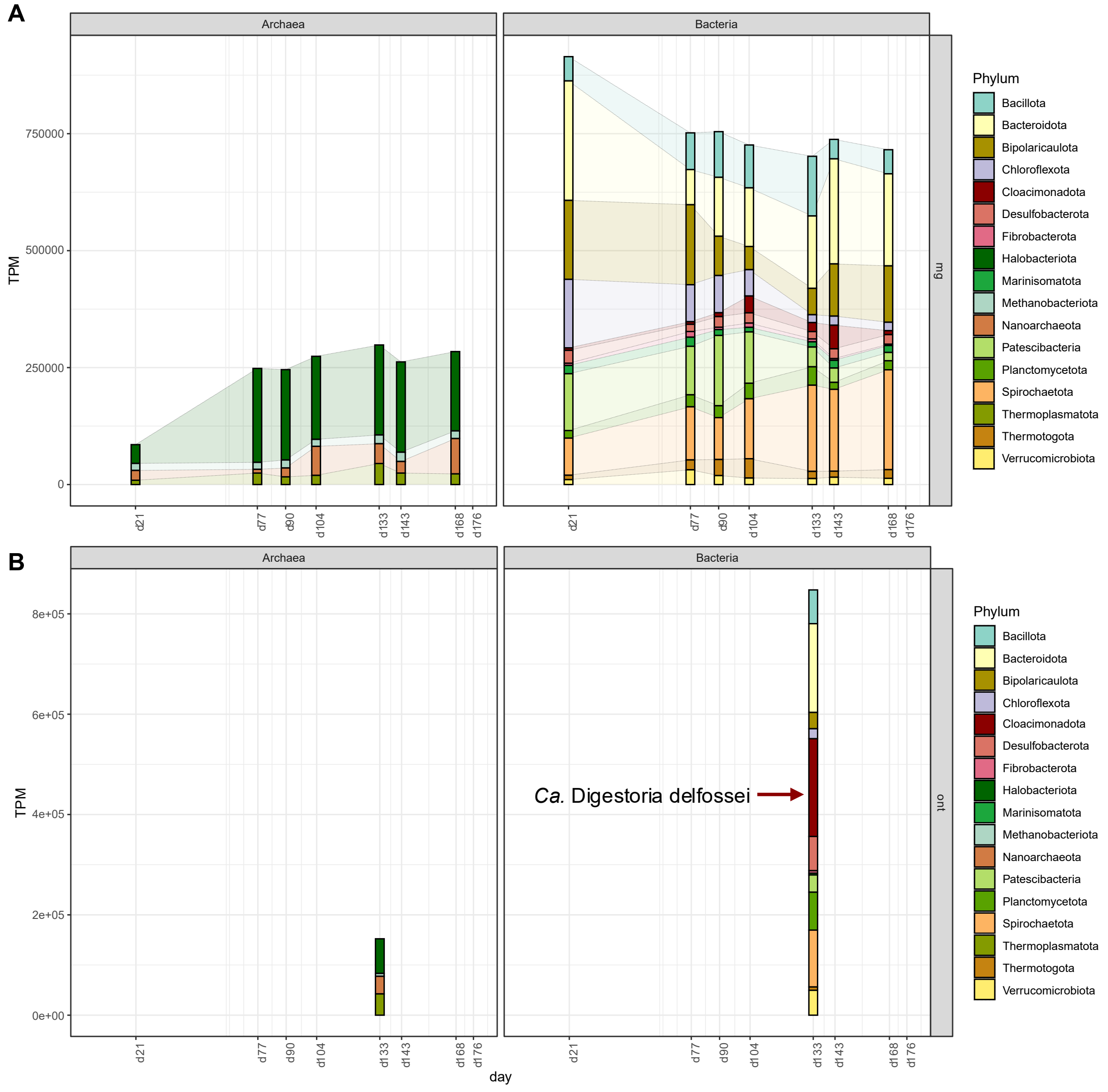

Figure S6. Phylum-level metagenomic abundance over time of metagenome-assembled genomes (MAGs) reconstructed in this study, based on short (A) and long (B) read sequencing. The genome of *Ca.* Digestoria delfossei, corresponding to OTU_1 in Fig. 1, is highlighted.

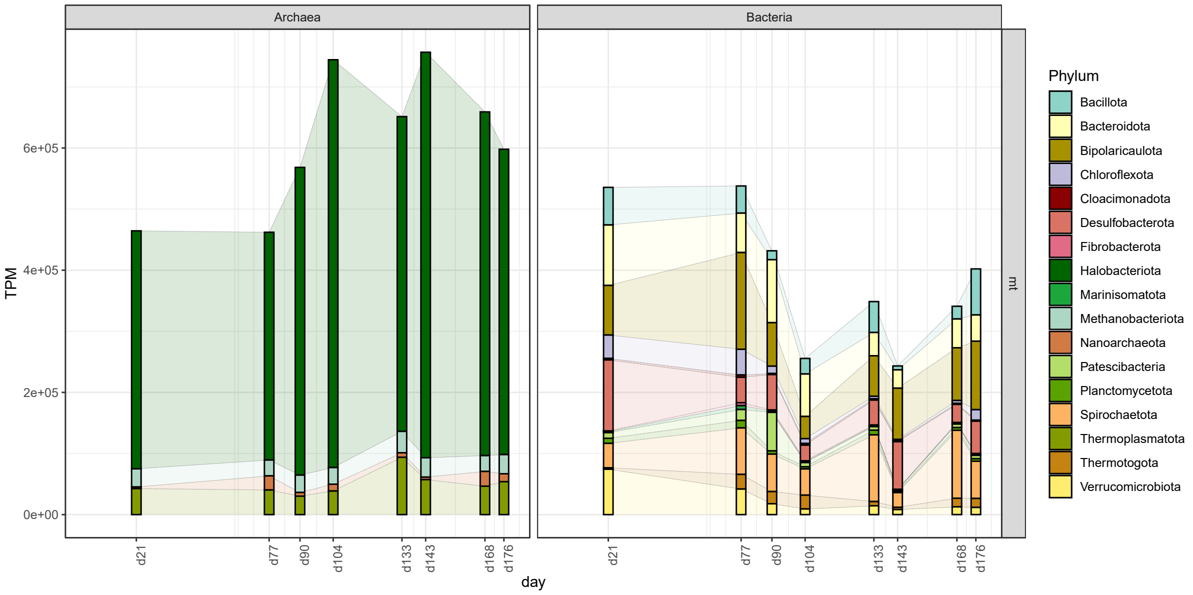

Figure S7. Phylum-level metatranscriptomic abundance over time of metagenome-assembled genomes (MAGs) reconstructed in this study (A). High metatranscriptomics abundance of archaeal MAGs results from very high gene transcript abundance of genes involved in the methanogenesis pathway (B).

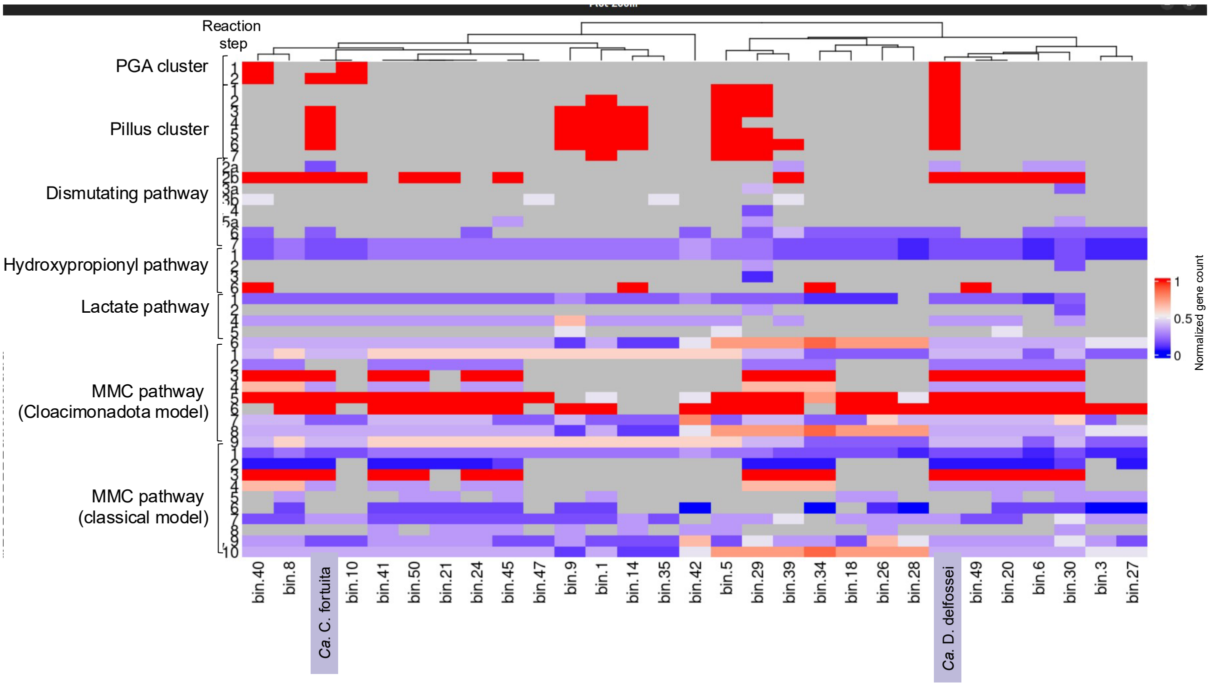

Figure S8. Potential syntrophic propionate-oxidizing bacteria (SPOB) based on pathway completion in the metagenome-assembled genomes generated in this study (Supplementary Material, Table S11).

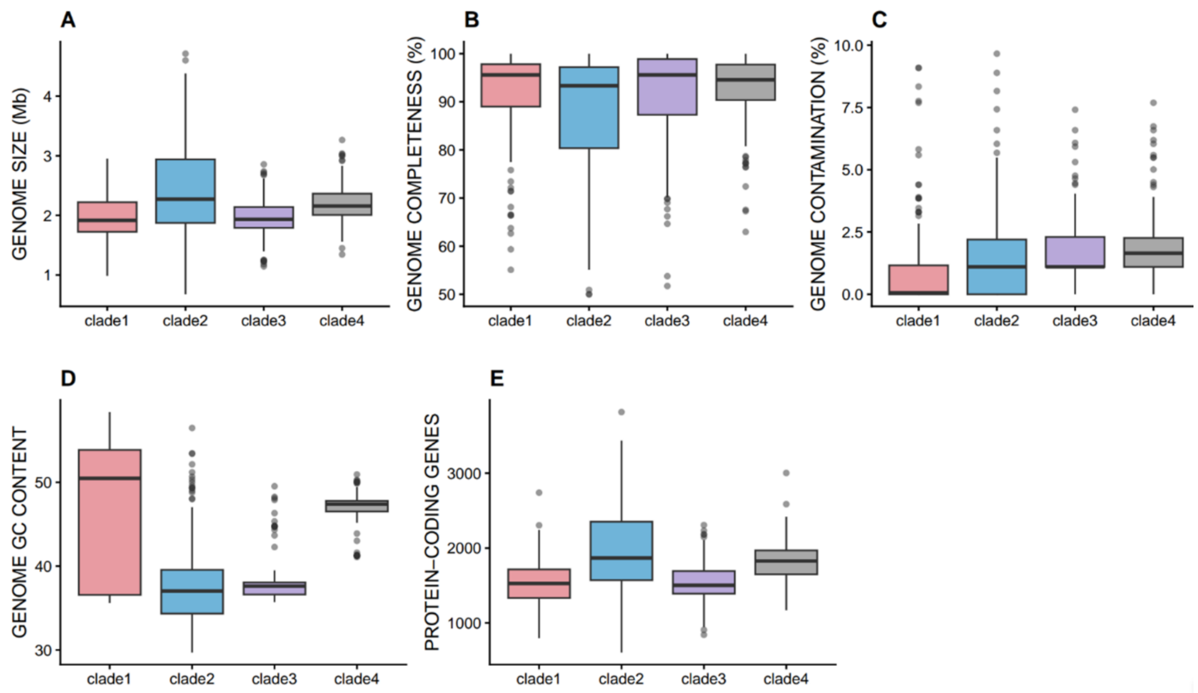

Figure S9. Characteristics of Cloacimonadota genomes used in the final database at the tree clade level (Fig. 2), including average genome completeness (A), average genome contamination (B), average GC content per genome cluster (C), average genome size per genome cluster (D), and number of protein-coding genes (E).

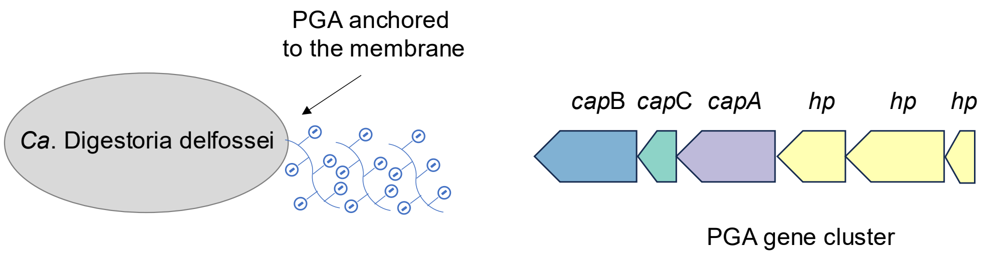

Figure S10. Gene organization in a poly-ɣ-glutamate (PGA) biosynthesis cluster within the *Ca*. Digestoria delfossei genome. PGA – poly-ɣ-glutamate; hp – hypothetical protein.

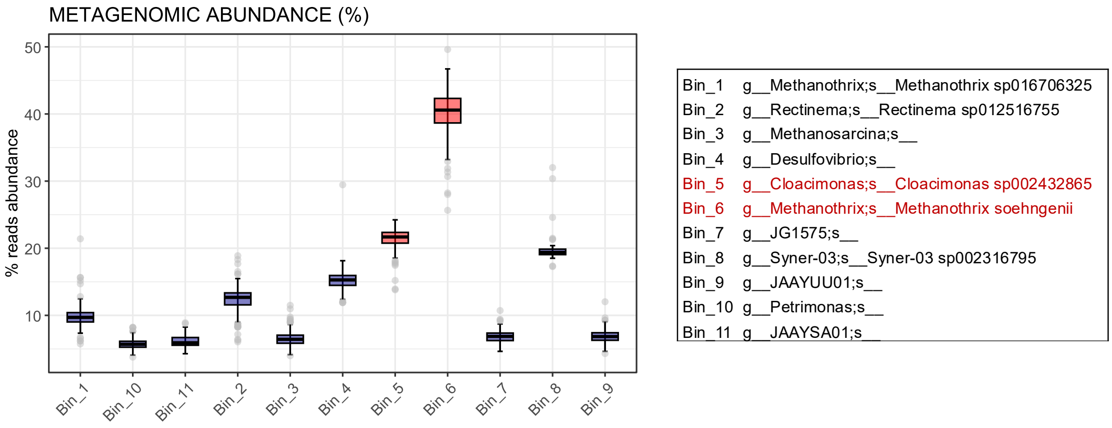

Figure S11. The metagenomic abundance (% of reads) of species (bins) identified in the Cloacimonadota-enriched culture grown on propionate (10g/L), along with their taxonomic classification using the GTDB-Tk database.

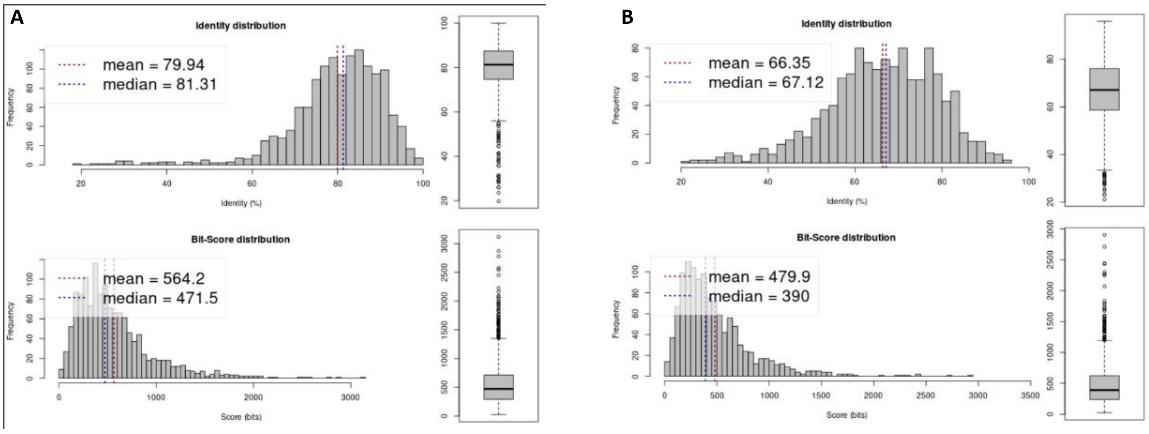

Figure S12. Amino acid identity (AAI) scores for shared proteins between *Ca*. Cloacimonas fortuita and *Ca*. Cloacimonas acidaminovorans (A) and *Ca.* Digestoria delfossei and *Ca.* Syntrophosphaera thermopropionivorans (B). The top panel shows the percentage identity distribution, indicating the level of amino acid similarity between the compared genomes. The bottom panel displays the bit-score distribution, reflecting the statistical significance of the alignments.

Description of Supplementary Datasets and Tables

Supplementary Dataset 1: Anaerobic baffled reactors (ABR), experimental design and 16S rRNA gene amplicon sequencing.

Table S1. Lab-scale anaerobic baffled reactors (ABRs) and measured variables.

Table S2. Count table of the 16S rRNA gene amplicon sequencing for bacteria. Reads were normalized to 8,000 per sample. Samples marked with * are the samples presented in Fig. 1.

Table S3. Count table of the 16S rRNA gene amplicon sequencing for archaea. Reads were normalized to 5,000 per sample. Samples marked with * are the samples presented in Fig. S3.

Table S4. Topological features for ASVs involved in the direct "Cloacimonadota OTU_1" (*Ca*. Digestoria delfossei) neighbor correlation network for the dataset of this study merged with the dataset from Lemaigre et al., 2018.

Supplementary Dataset 2: Genome reconstruction of microbes from Anaerobic Baffled Reactor 1 (ABR1) and the Cloacimonadota genome database.

Table S5. Characteristics of metagenome-assembled genomes (MAGs) reconstructed in this study from Illumina short-read metagenomics (before genome refinement for bin36 representing the *Ca*. Digestoria delfossei).

Table S6. Details of the two reconstructed Cloacimonadota genomes from species enriched in this study.

Table S7. Complete list of 58 Cloacimonadota metagenome assembled genomes (MAGs) used in the final Cloacimonadota database, including MAGs generated in this study and from other sources (downloaded in July 2025).

Table S8. Characteristics of KEGG orthologues (KO) assignments in genomes included in the final Cloacimonadota database. The numbers indicate the presence of specific KOs in genomes belonging to the Cloacimonadota clade (as shown in Fig. 2). Maaslin2 v1.22.0 was employed to identify features (i.e., KOs) that best explain differences between AD and aquactic/marine and freshwater clades.

Table S9. Protein clusters (PCs) generated by MMSeq2 (Mirdita et al., 2019) and their functional assignment to KEGG orthologues (KOs) for the Cloacimonadota genomes included in the final database (Table S7).

Table S10. Summary of the carbohydrate-active enzymes (CAZy) domains identified by dbCAN2 (Zhang et al., 2018) for the Cloacimonadota genomes included in the final database (Table S7) and summarized at the tree clade level (Fig. 2).

Supplementary Dataset 3: Syntrophic propionate oxidation (SPO) pathway and supporting results

Table S11. KEGG orthologues (KOs) assignments to the various SPO pathways analyzed in this study. The hydroxypropionyl and lactate SPO pathways are adapted from Paton et al., 2020. Details on the alternative mmc pathway proposed for Cloacimonadota are given in Table S12.

Table S12. Reaction steps and enzymes involved in the proposed alternative mmc pathway in Cloacimonadota.

Table S13. Genes associated with KOs involved in the steps of syntrophic propionate oxidation pathways and assigned to the MAGs generated in this study (Table S5). Gene expression levels are presented as TPMs, with raw read counts also included.

Table S14. Proteins identified through peptides from the metaproteomic study, involved in the mmc pathway (Cloacimonadota alternative) or associated with SPO, are highlighted. The search was performed against the complete genomes of the newly reconstructed *Ca.* Digestoria delfossei and Methanothrix bin.28.

Supplementary Dataset 4: Distinct and overlapping metabolic capacities of Cloacimonadota compared to other phyla in anaerobic digestion (AD) reactor

Table S15. Representation of KEGG orthologues (KOs) in the genomes of Cloacimonadota (Table S7; limited to genomes from AD microbes) and other microbes within the AD microbiome (Campanaro et al., 2020). Only genomes with at least 70% genome completeness are included.

Supplementary Dataset 5: Enrichment and cultivation trials of Cloacimonadota.

Table S16. Summary of the conditions used to enrich *Ca.* Cloacimonadota from anaerobic digestion sludge (first stage). In bold, the culture with significant enrichment of *Ca.* Cloacimonas fortuita is shown.

Table S17. Screening of enriched cultures for the presence of novel Cloacimonadota species, with results expressed as the percentage of read abundance based on the 16S rRNA gene amplicon sequencing.

Table S18. Summary of the conditions used to enrich Ca. Cloacimonas fortuita from the RT_BT_as_ph7 culture initially enriched during the first stage (Table S17).

Table S19. List of species identified in the Cloacimonetes-enriched culture (*Ca.* Cloacimonas fortuita) grown RT_BT_as_ph7 on propionate (Clo3, Table S18) along with their genome characteristics. Genomes were reconstructed from short Illumina reads. *Ca.* Cloacimonas fortuita bin is highlighted in bold.
